# The formation of spinel-group minerals in contaminated soils: the sequestration of metal(loid)s by unexpected incidental nanoparticles

**DOI:** 10.1186/s12932-019-0061-3

**Published:** 2019-03-13

**Authors:** Michael Schindler, Haley Mantha, Michael F. Hochella

**Affiliations:** 1 Department of Earth Sciences, Laurentian University, Sudbury, ON Canada; 2 Department of Chemistry, Laurentian University, Sudbury, ON Canada; 3 Department of Geosciences, Virginia Tech, Blacksburg, VA 24061 USA; 4 Subsurface Science and Technology Group, Pacific Northwest National Laboratory, Richland, WA 99352 USA

**Keywords:** Spinels, Contaminated soils, Pedogenic, Mineral nanoparticles, Mineral surface coatings, Organic matter, Low temperature

## Abstract

Mineralogical studies of contaminated soils affected by smelter emission and dust from mining activities indicate that minerals of the spinel group are one of the common hosts of metal-bearing contaminants. Spinel group minerals typically originate from high temperature processes, but an increasing number of studies indicate that metal-bearing spinel group minerals can also form under ambient Earth surface conditions in surficial soils. In this contribution to honor Donald Sparks, we show that the spinels Zn-bearing magnetite (Zn_0.5_Fe_2.5_O_4_) and minium (Pb_3_O_4_) form during low temperature alteration of Pb-bearing silica glass in surficial organic rich soils in proximity to a former Cu-smelter in Timmins, Ontario, Canada. The glass most likely formed during high-temperature processes and has been either emitted by the smelter or wind-blown from waste rock piles to near-by soils. The alteration of the glass by percolating pore solutions has resulted in the formation of large micrometer-size dendritic etch features and in nanometer-size dendritic alteration halos composed of nano-size prismatic crystals of Zn-rich magnetite and spherical nanoparticles of minium. Both spinel-type phases are embedded in an amorphous silica matrix which formed during the alteration of the glass at low temperature. A review on the occurrence of spinel-group minerals in smelter-affected soils or mine tailings indicates that the formation of these minerals under ambient Earth surface conditions is quite common and often results in the sequestration of contaminants such as Cu, Ni, Zn and Sb. The pedogenic spinels often occur as euhedral crystals in nano-size mineral assemblages within alteration features such as dendritic etch patterns, mineral surface coatings and mineralized organic matter. Their well-developed crystal forms indicate that (a) they have not formed during a rapid cooling process in a smelter or refinery which typically creates spherical particulate matter, and (b) they have not been part of particulate matter added via fluvial or Aeolian processes which most commonly yield anhedral morphologies. The formation of nano-size spinel-group minerals in low temperature environmental settings may lead to the long-term storage of metal(loid)s in mineral phases and their transport over vast distances via fluvial, alluvial and Aeolian processes.

**Electronic supplementary material:**

The online version of this article (10.1186/s12932-019-0061-3) contains supplementary material, which is available to authorized users.

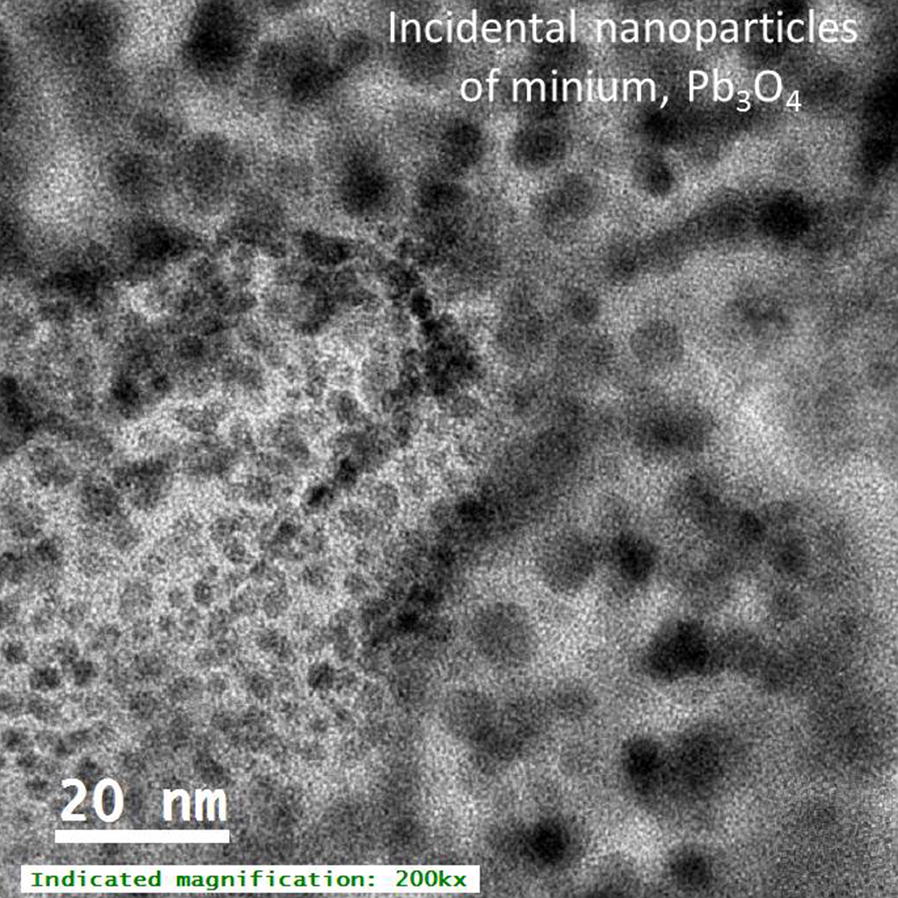

## Introduction

Incidental nanoparticles (nanomaterials unintentionally produced as a result of any form of direct or indirect human influence or anthropogenic process) are common in the most affected areas of Earth, including in and around manufacturing facilities, mining areas, power plants, waste water treatment plants, agricultural lands, and surface and subsurface waters associated with all of these areas [1, 2]. Knowledge about the formation and chemical and physical interactions of incidental nanoparticles with their surroundings is very often necessary to understand the fate of pollutants in the environment. These nanoparticles are known to either structurally incorporate metal(loid) contaminants from their immediate environment via adsorption or absorption processes, and potentially transport them over vast distances via fluvial, alluvial and Aeolian processes [3, 4].

Mineralogical studies of soils affected by emissions from smelters and refineries or by dust windblown from nearby or far-away mining activities indicate that minerals of the spinel group are one of the major hosts of metal contaminants [5, 6]. We suspect that mineralogical studies of affected soils have overlooked the sequestration of contaminants by pedogenic minerals of the spinel-group, such as magnetite, because they were thought not to form under ambient Earth surface conditions [7]. In addition, finding and studying minerals in the nano-scale range is expensive and difficult. In this study to honor the achievements of Donald Sparks, we will show that euhedral prismatic crystals of Zn-rich magnetite, and nanoparticles of the mineral minium (Pb_3_O_4_), both from the spinel group, form during low-T alteration of Pb-bearing silica glass in the contaminated soils around the former Kidd Creek smelter complex in Timmins, Ontario, Canada. The occurrence of these pedogenic spinel phases will be compared with previous observations on the formation of spinel group minerals during low-T abiotic and biotic-controlled alteration processes in mineral surface coatings and mineralized organic matter.

## Background information on spinel-group minerals

Minerals and phases of the spinel group are of great environmental, geological and industrial importance as they can incorporate a large variety of di-, tri-, tetra- and pentavalent cations [8]. The general formula of simple spinels is *AB*_2_O_4_ where the divalent *A* cation can either occupy a tetrahedral- (“normal” spinel) or octahedral-coordinated site (reverse spinel) in the spinel structure.

Many spinel group minerals have limited geological occurrences, but spinel-group minerals containing Zn, Cr, Fe and Ti are abundant enough to be considered important ore minerals. These spinel ore minerals include willemite, Zn_2_SiO_4_ and chromite, FeCr_2_O_4_. Iron-rich spinels such as magnetite Fe_3_O_4_, maghemite, Fe_2_O_3_ and titanomagnetite, FeTiO_4_, are common and occur in igneous massifs as cumulate strata, in metamorphic terrain and in sedimentary deposits either as refractory minerals or in association with lateritic-type deposits.

The upper horizons of many modern and buried soils have higher concentrations of ferrimagnetic minerals such as magnetite and maghemite than the parent material from which they were originally derived [10]. Lithogenic magnetite is a common mineral in these soils as part of the coarse, heavy mineral fraction. The occurrence of pedogenic magnetite has only been established since the late 80’s as researchers showed that magnetite can form through abiotic [7] and biotic [11] processes in soils. Pedogenic magnetite occurs commonly in the nano-size fractions of soils and is a common reduction product of ferric oxyhydroxides in microaerobic and anaerobic sediments and soils [9, 10, 12]. The biotic formation of spinels typically involves biomineralisation by Fe-reducing bacteria which gain energy through oxidation of organic matter using nitrate and Fe(III). These bacteria can be magnetotactic such as *Aquaspirillum magnetotactum* or reducing such as *Geobacter sulfurreducens* and *Shewanella oneidensis* [9, 11].

Abiotic formed magnetite NPs can form by co-precipitation reactions that, as shown in analogous synthesis experiments, follow a number of different pathways. However, the predominant process is a topotactic transformation of goethite to magnetite [12]. Nucleation and growth of abiotic magnetite NPs can also precede through rapid agglomeration of spheroidal Fe-hydroxide nanoparticles particles (5–15 nm in diameter) without the involvement of an amorphous bulk precursor phase [13]. In aerated environments magnetite is unstable and is subject to weathering. Maghemite is the main weathering product of magnetite. Maghemite nanoparticles in soils can also form through dehydration of lepidocrocite nanoparticles [12].

## Methodology

### Background information on the Kidd Creek metallurgical site

**Fig. 1:**
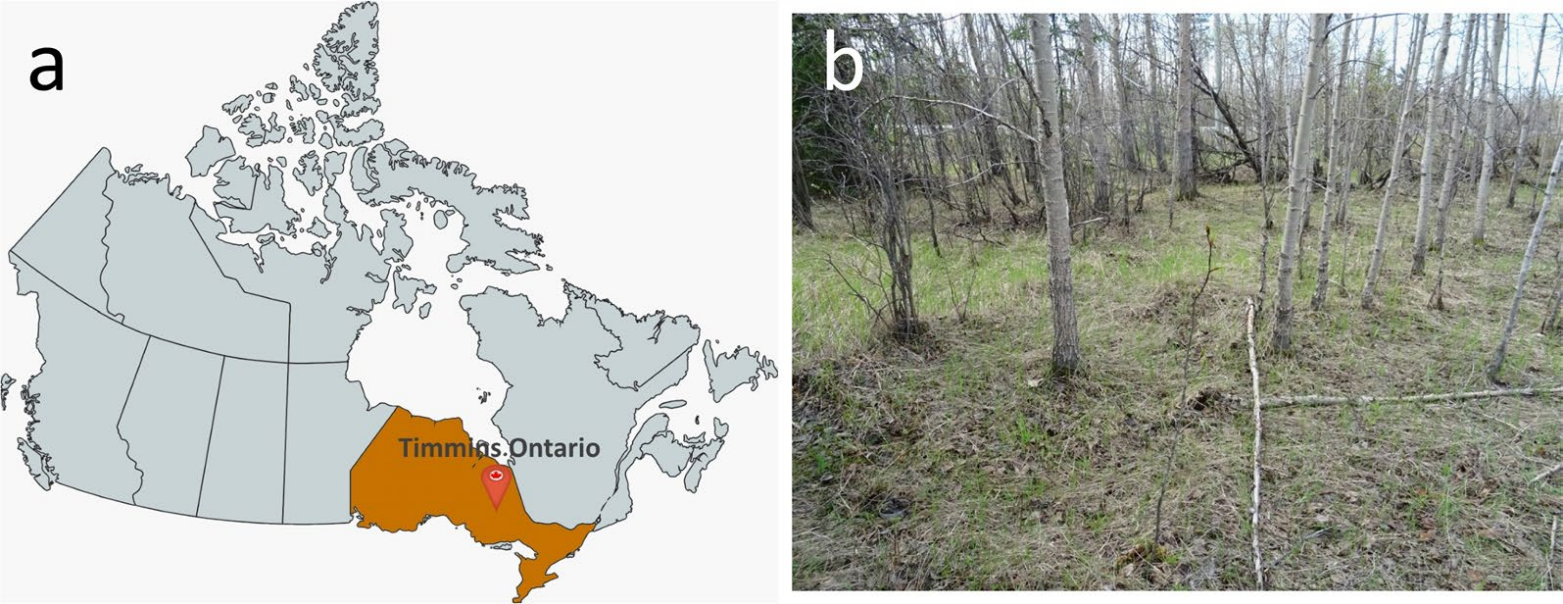
**a** Map of Canada indicating the location of Timmins, Ontario with a red point symbol; **b** photographic image of the sampling location, circa 1.3 km from the former Kidd Creek smelter complex

The Kidd Creek metallurgical site, located within the city limits of Timmins, Canada (Fig. 1a), was in operation for 30 years, closing in 2010. The ore processed at this location contained predominantly chalcopyrite (CuFeS_2_), pyrite (FeS_2_), bornite (Cu_5_FeS_4_), pyrrhotite (Fe_(1−x)_S_x_ = 0–0.2), sphalerite (ZnS), and galena (PbS) [14]. The ore was shipped via train from the mine site to the metallurgical site for processing (27 km away). The Mitsubishi copper smelting process was employed at start-up with furnaces operating around 1200–1300 °C [15]. From 2002 to 2009, the average amount of total particulate matter (PM < 100 μm) released to the atmosphere was 844 t/year [16].

### Sample collection, preparation and characterization

A detailed description of sampling procedures and preparation techniques of soil samples collected at various sites around the Kidd Creek smelter complex is given in Mantha et al. [17]. Here we only describe the methodology for the sampling and characterization of the Pb-bearing silica glass. Surficial soil samples (~ upper 5 cm) were collected in October 2016 from a site in close proximity to the former smelter complex within a birch stand with a grassy understory (Fig. 1b). The sample was transported to the lab in a cooler, dried at 80 °C, sieved (< 1.4 mm), and stored under dry conditions in sealed bags.

#### Scanning electron microscopy, focused ion beam technology and transmission electron microscopy

**Fig. 2:**
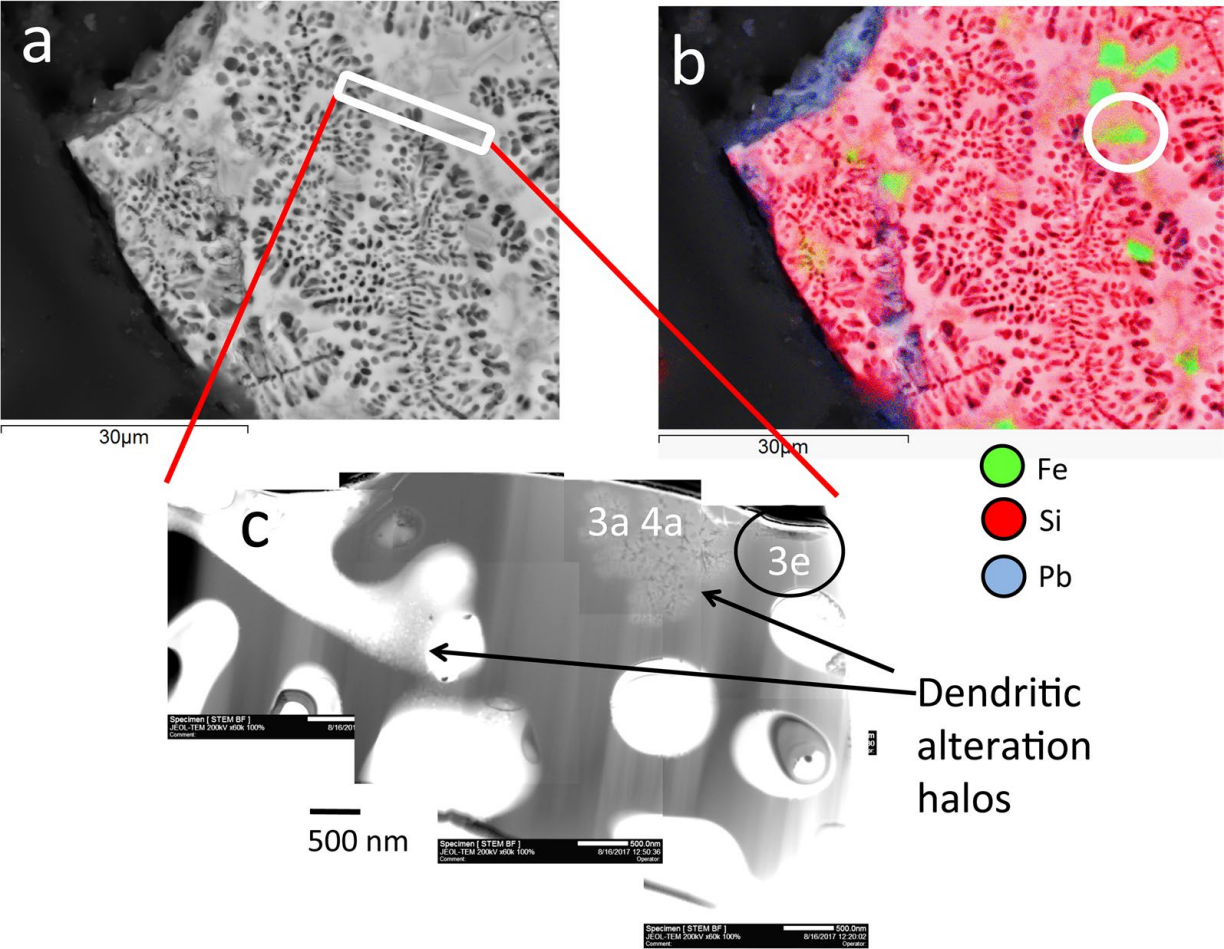
SEM images in Backscattering Electron mode (BSE) of a selected area of the Pb-bearing glass grain indicating the dendritic etch features (black) within the glass matrix (white); the location of the extracted FIB section is indicated with a white rectangle; **b** image combining the BSE image shown in **a** with SEM–EDS chemical distribution maps for Fe (green), Pb (blue) and Si (red); areas depicted in lighter pink represent the unaltered Pb-silicate glass matrix whereas areas in darker pink depict Si-enriched alteration areas; the location of the remnants of a Zn-rich magnetite crystal in the extracted FIB section is encircled; **c** scanning TEM (STEM) images in bright field mode (BF) of the entire extracted FIB section; the orientation of the FIB section relative to the area from which it was extracted is indicated with red lines; the remnants of a Zn-rich magnetite crystal shown in **b** is encircled; the location of the areas shown in the Figs. 3a, e and 4a are labelled accordingly

The Pb-bearing glass sample was first characterized using backscattering secondary electron and energy dispersive spectroscopy imaging with a Zeiss Evo 50 Scanning Electron Microscope (Geoscience laboratories, Sudbury, Ontario) operating with an accelerating voltage of 20 kV and a beam current of 750 pA. A specific area in the cross section of the glass grain was subsequently selected for extraction of a focused ion beam (FIB) section with a FEI Helios 600 NanoLab FIB (Fig. 2a, b). The section was subsequently lifted using a platinum gas-glue, thinned to electron transparency by ion gas milling (Ga^+^ ions) and mounted on a molybdenum holder. Transmission electron microscopy (TEM) was conducted with a JEOL 2100 transmission electron microscope (a field thermionic emission analytical electron microscope) at the Virginia Tech National Center for Earth and Environmental Nanotechnology Infrastructure (NanoEarth). Measurements were taken with an accelerating voltage of 200 kV and a beam current of approximately 107 mA. EDS point analyses and maps were acquired in STEM mode with JEOL bright field (BF) and JED-2300T EDS detectors. Selected area electron diffraction (SAED) patterns were acquired using a Gatan Orius SC200D detector. Nanoparticles and larger crystals were identified using a combination of SAED, fast Fourier transformations (FFT) of lattice fringes, and EDS-STEM chemical distribution maps.

## Results

The surficial upper 0–5 cm of the collected soils are brownish black, oxic and slightly acidic with a pH/Eh of 5.0/726 mV [18]. They contain 2.1 wt% Fe, 1.3 wt% Cu, 0.8 wt% Pb and 685 mg kg^−1^ Zn [18]. The coarse sand size fraction (> 180 μm) of the surficial soil layer is composed predominantly of organic matter with minor amounts of quartz (SiO_2_) and traces of digenite (Cu_9_S_5_), talnakhite (Cu_9_(Fe, Ni)_8_S_16_), and magnetite (Fe_3_O_4_) [18]. The finer silt to sand size fraction (< 180 μm) contains mainly quartz (SiO_2_), hematite (Fe_2_O_3_) and minerals of the feldspar group with minor silicified organic grains, spherical smelter-derived particulates and angular silicate-based particles [18]. Interaction of the sulfide-rich particulate matter with the organic matter and the speciation of Cu within organic residues are described in Mantha et al. [18]. In this study, we focus on the formation of low-T alteration products in pore spaces of a Pb- bearing silica glass grain identified in the coarser fraction (> 180 μm).

### Chemical and textural features of the Pb-bearing silica glass grain

The cross section of the angular glass grain has the dimension ~ 350 × ~ 200 μm. The cross-section is greyish-white and contains a prominent red-coloured alteration rim (Additional file 1: Figure S1). The glass is predominantly composed of Si, Pb, and O with minor Al, Fe, Cu and Zn (Additional file 1: Figures S2, S3). Assuming that Fe, Cu and Pb occur predominantly in their tri- and divalent states, the average composition of the glass is (K_0.01_, Ca_0.02_ Al_0.02_Cu_0.02_Zn_0.04_Fe_0.10_Pb_0.25_Si_0.72_O_2_) (n = 5).

Dendritic etch features occur throughout the glass grain creating rounded pore spaces with diameters up to 2 μm (Fig. 2d). The glass matrix also contains large micrometer-size Zn-rich magnetite crystals with maximum Zn:Fe atomic ratios of 1:5 (green in Fig. 2c).

**Fig. 3:**
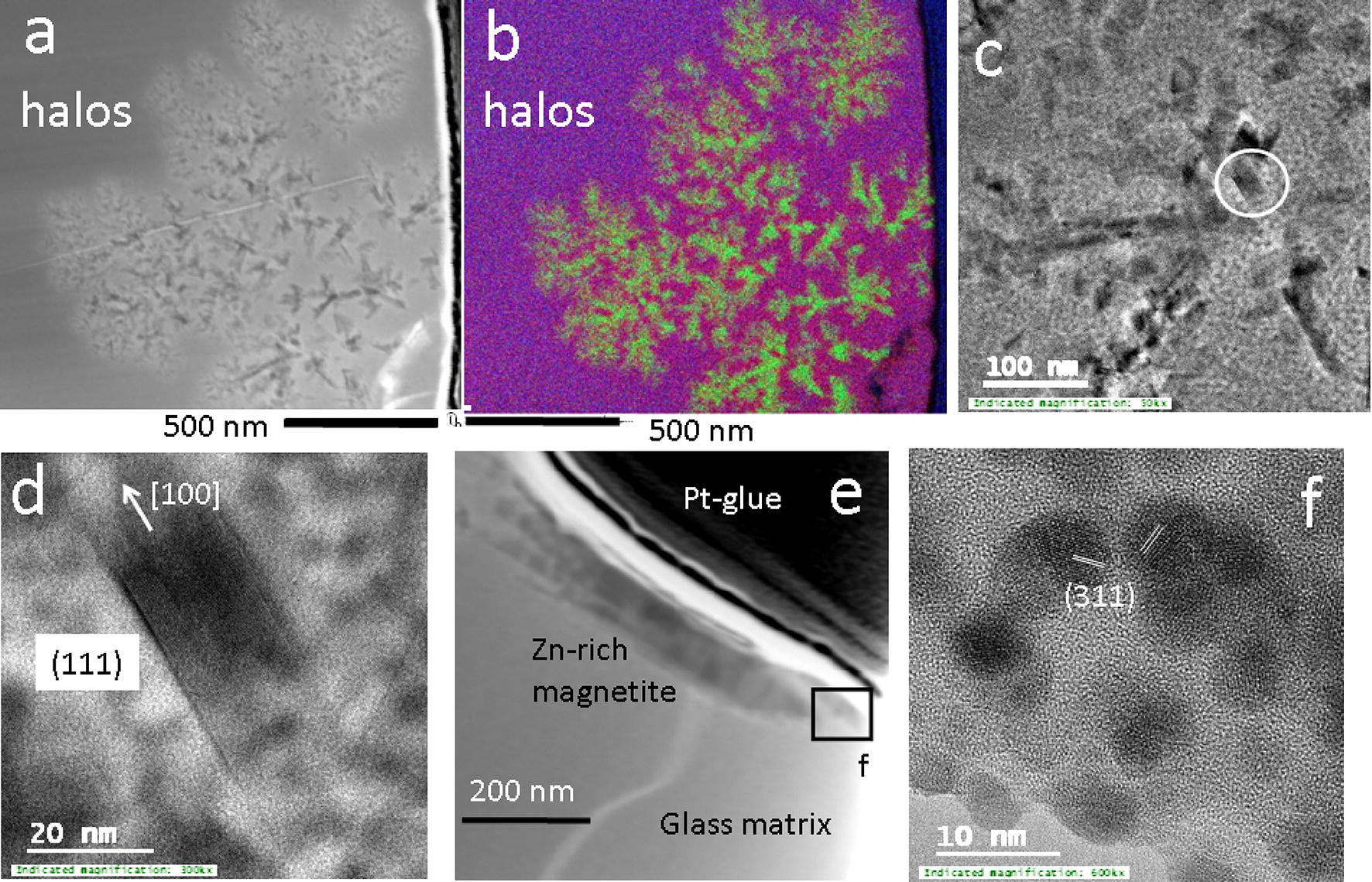
**a**, **b** STEM-BF image and chemical distribution maps for Fe (green), Pb (blue) and Si (red) of an alteration halo containing dendritic growth/dissolution features; **c** TEM image of latches of Zn-rich magnetite within the branches of the dendrites; the location of the crystal shown in **d** is encircled; **d** high-resolution TEM image of a Zn-rich magnetite crystal; a predominant crystal face and the direction of growth are labelled accordingly; **e** remnants of a micrometer size Zn-rich magnetite crystal along the surface of the FIB towards the Pt-glue; the area shown in **f** is indicated with a black square; **f** agglomeration of spherical Zn-rich magnetite nanoparticles along the rims of the latter crystal; orientation of the lattice fringes parallel to (311) are highlighted with white lines

The FIB section was extracted along the interface between an area containing dendritic etch-features and a Zn-rich magnetite crystal (encircled; Fig. 2). The FIB section is composed of a highly porous glass matrix, the remnants of a Zn-rich magnetite crystal (${\text{Zn}}_{0.5} {\text{Fe}}_{0.5}^{2 + } {\text{Fe}}_{2}^{3 + } {\text{O}}_{4}$) with traces of goethite and a dendritic alteration halo (Fig. 2c, Additional file 1: Figures S4, S5). The branches of the latter halo contain lathes of Zn-rich magnetite (${\text{Zn}}_{0.5} {\text{Fe}}_{0.5}^{2 + } {\text{Fe}}_{2}^{3 + } {\text{O}}_{4}$) (Fig. 3a and in green in Fig. 3b, Figures S6–S8) and nanoparticles of minium (Pb_3_O_4_) (Additional file 1: Figures S9, S10). These phases are embedded in a matrix enriched in Si (in pink) relative to the glass matrix (in violet) (Fig. 3a–c and Additional file 1: Figure S11). High resolution TEM images indicate that the Zn-rich magnetite lathes are agglomerates of elongated prisms with prominent (111) faces growing parallel to [100] (Fig. 3c, d). Contrary, the remnant of the larger Zn-rich magnetite crystal occurs in a relative homogenous glass matrix lacking an alteration halo (Fig. 3e). The outer rims of the crystal are composed of agglomerated spherical nanoparticles displaying (311) lattice planes in different orientations (Fig. 3f).

**Fig. 4:**
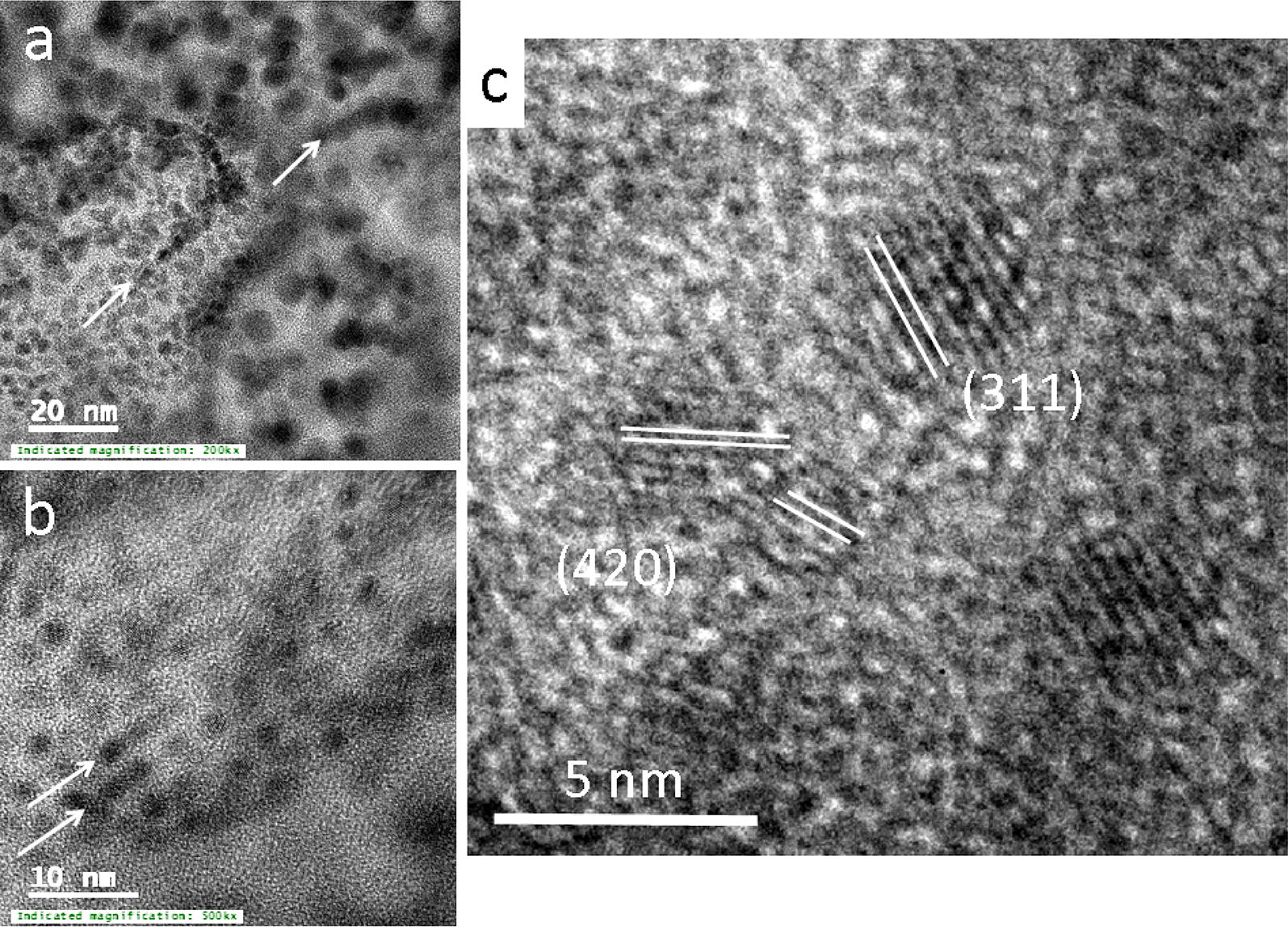
**a**, **b** TEM images depicting the agglomeration of minium nanoparticles to linear or curvilinear features (labelled with arrows); **c** high resolution TEM images of minium nanoparticles in a silica matrix; lattice fringes parallel to (311) and (420) are highlighted with white lines

Minium commonly forms in the vadose zone of Pb-ore deposit as a result of the oxidation of galena, PbS. The presence of Pb^4+^ in its structure and its prominent red colour can be used as optical indicators for the degree of oxidation of Pb in the vadose zone [19]. The minium nanoparticles in the silica-rich matrix were identified on the basis of (a) d-spacings observed in SAED and FFT pattern (d = 2.87 Å (112), d = 2.61 Å (202), d = 2.25 Å (311) and d = 2.0 Å (420)), (b) a higher abundance of Pb in the nanoparticles than in the surrounding (hydrous) silica matrix and (c) the observation that a red-coloured alteration rim occurs along the glass grain (Additional file 1: Figure S1). The diameter of the spherical minium nanoparticles varies between 2 and approximately 5 nm (Fig. 4a–c). In areas of high nanoparticle density, the particles agglomerate to linear or curvilinear features (indicated with white arrows in Fig. 4a, b). Nanoparticles depicting the same type of lattice fringes appear to attach to each other in an orientated fashion as their lattice fringes depict similar orientations (Fig. 4c).

## Discussion

Silicate-based glasses are common in smelter-affected soils [20–22]. They commonly form during rapid cooling of the slag or from hot droplets in the smelter stack. The Pb-bearing silica glass examined in this study most likely formed during one of these cooling processes and was released into the environment through either smelter emissions or Aeolian transport from mine waste piles. Similar to volcanic glass, smelter-derived silicate-based glasses are more susceptible to weathering than their crystalline counterparts [20, 21]. This is also evident in the case of the Pb-bearing silica glass which depicts an extensive three-dimensional network of dendritic etch features (Fig. 2a–c).

### Origin of the dendritic etch and growth features

Dendritic growth and dissolution patterns are not geometrically related to the crystal structure of the precipitating or dissolving phase. The patterns are characterized by random dendritic fractal formed by an irreversible precipitation or dissolution process occurring in conditions far from equilibrium [23]. Dendritic growth and dissolution patterns are favoured when the kinetics of a precipitation or dissolution process at the fluid–solid interface is fast, but the resulting growth or dissolution process is limited by the diffusive transport of solutes or solvents to the interface [24]. Hence, the observed dendritic dissolution features in the Pb-bearing silica glass formed as a consequence of the fast dissolution kinetics of the glass and limited diffusion of the solvents to the interface and dissolution products from the interface to the bulk soil.

The growth of the Zn-rich magnetite and formation and agglomeration of minium nanoparticles was however not controlled by the diffusion of Zn-, Fe- and Pb- bearing species or nanoparticles from the bulk soil to the interface, as all three elements occurred in the glass matrix and were released during its dissolution. Their formation was most likely a result of a mineral replacement reaction where Pb-bearing silica glass (light pink in Fig. 2b, violet in Fig. 3b) was replaced by (most likely hydrous) amorphous silica along the reaction front (dark pink in Fig. 2b and 3b and black in Fig. 3a). The formation of silica-enriched surface layers during the alteration of silica-based glass is a common alteration feature and has been explained with a dissolution–precipitation or leaching mechanism [25]. These silica-rich alteration layers can be highly porous and allow a mass exchange between the infiltrating solutions and the reaction front on the surface of the unaltered glass [24]. In the case of the alteration of the Pb-bearing silica glass, released Zn, Fe and Pb-bearing species diffused from the reaction front through the porous silica layer towards the branches of the dendrites, which resulted in the observed Si-enriched areas between the branches and the reaction front. A limited mass exchange between the alteration halo and the bulk soil led to the accumulation of Zn, Fe and Pb within dendritic branches and in the subsequent formation of Zn-rich magnetite nano-crystals (in green in Fig. 3b and black in Fig. 3a, c and d) and minium nanoparticles (Fig. 4a–c). Agglomeration of the minium nanoparticles in an orientated fashion (Fig. 4c) led first to linear and curvilinear linear aggregates (Fig. 4a, b) and subsequently to larger micrometer-size red-coloured alteration rims (Additional file 1: Figure S1).

The dendritic alteration halo may reflect an early stage of the dendritic dissolution pattern as individual halos have approximately the same size and shape as the etch features in the dissolution pattern (Fig. 2c). Hence it seems likely that the replacement of the glass by (hydrous) amorphous silica, Zn-rich magnetite and aggregates of minium occurred whenever a percolating solution initiated the alteration of the glass. The subsequent removal of these alteration products was likely a result of an increase in the fluid-rock ratio within the micrometer-size etch features (Fig. 2).

The much larger micrometer-size Zn-rich magnetite crystals in the glass matrix formed most likely through a different process than their nanometer size counterparts (Figs. 2b, 3e). This conclusion is supported by the absences of Si-rich alteration halos and minium nanoparticles around the remnants of the micrometer Zn-rich magnetite crystal (Fig. 3e) which would have formed during low-T alteration of the glass. The micrometer size Zn-rich magnetite crystal is instead surrounded by spherical magnetite nanoparticles which agglomerate to larger aggregates along its rim (Fig. 3f). Furthermore, idiomorphic magnetite crystals with similar size as those observed in the Pb-bearing silica glass (Fig. 2b) are common features in silicate-based glass matrices within smelter-derived spherical particulates [19, 20]. These observations indicate that the micrometer-size Zn-rich magnetite crystals in the matrix of the Pb-bearing silica glass crystallized during formation of the glass at high T through the attachment of nanoparticles to a growing crystal surface. The latter type of crystallization mechanisms is commonly referred to as crystallization by particle attachment (CPA) [26].

The occurrence of two different types of Zn-rich magnetite crystals in the Pb-bearing silica glass is a good example of how to distinguish spinels formed during low- and high-T processes on the basis of textural and mineralogical features. Although both spinels occur in the form of euhedral crystals, the low-T form occurs in a highly porous environment in association with other low-T formed minerals, whereas the high-T spinel are embedded in an unaltered glass matrix formed at high-T.

This study could not unequivocally identify franklinite, ZnFe_2_O_4_, in the altered parts of the glass as well as in the bulk soil matrix. However, there should be no reason for the absence of this mineral as the Zn:Fe ratio in the unaltered glass is close to 1:2. In addition, franklinite has also been observed in the form of nano-size crystals in association with other low-T alteration products (see below) and as micrometer-size particulate matter in soils affected by smelter emissions [27].

### Other examples of spinels involved in the sequestration of metal(loid)s

Studies of nano-scale features in soils have predominantly focused on the occurrence and formation of clay-size minerals in order to gain an understanding of fundamental soil-forming processes [28]. On the contrary, the fate of contaminants in soils has been predominantly characterized with bulk analytical methods such as submicron resolution synchrotron-based spectroscopies or sequential extraction techniques [29, 30]. The site-specific extraction of micrometer-size ultra-thin sections with FIB and subsequent TEM studies with better than nanometer resolution now allows the investigation of mineralogical features in confined pore spaces of low-T alteration products such as mineral surface coatings and mineralized organic matter [31–35]. The lack of the latter studies in the past and the fact that bulk analytical techniques cannot distinguish between spinels formed during low and high T processes may explain why nano-size crystals of spinel group minerals in low-T alteration products are a formerly unknown feature in soils.

Below, we briefly review the occurrence of nano-size crystals of spinel group minerals in low-T alteration products within soils from other locations including Sudbury, Ontario and Trail, British Columbia, Canada.

#### The occurrence of Ni-bearing spinels in mineral surface coatings from Sudbury, Ontario, Canada

**Fig. 5:**
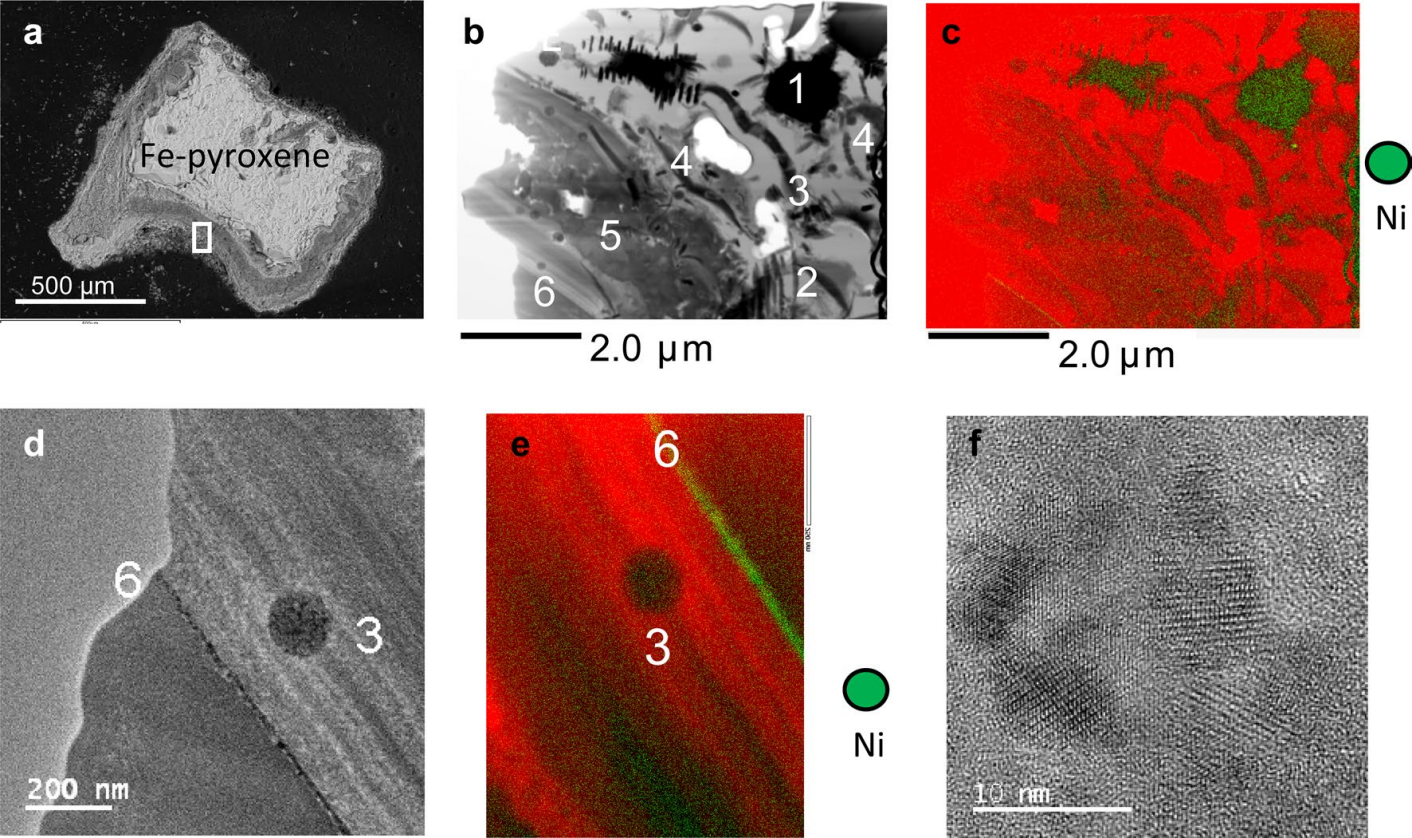
Spinel-type phases in a mineral surface coatings of the Sudbury area; **a** selected coatings on a Fe-rich pyroxene; the location of the extracted FIB section is indicated with a white-framed rectangular; **b**–**e** STEM and EDS-STEM images of selected areas in the extracted FIB sections; areas enriched (green) and depleted in Ni are numbered as follows: (1) magnetite cubes; (2) maghemite needles, (3) spherical magnetite NPs, (4) magnetosomes; (5) jarosite; (6) linear alignment of trevorite NPs between two chemically distinct zones in the coatings; **f** nano-crystals of trevorite displaying different orientations; a red background colour was chosen in the images **c** and **e** in order to highlight the occurrence of Ni in the spinel-type phases

**Fig. 6:**
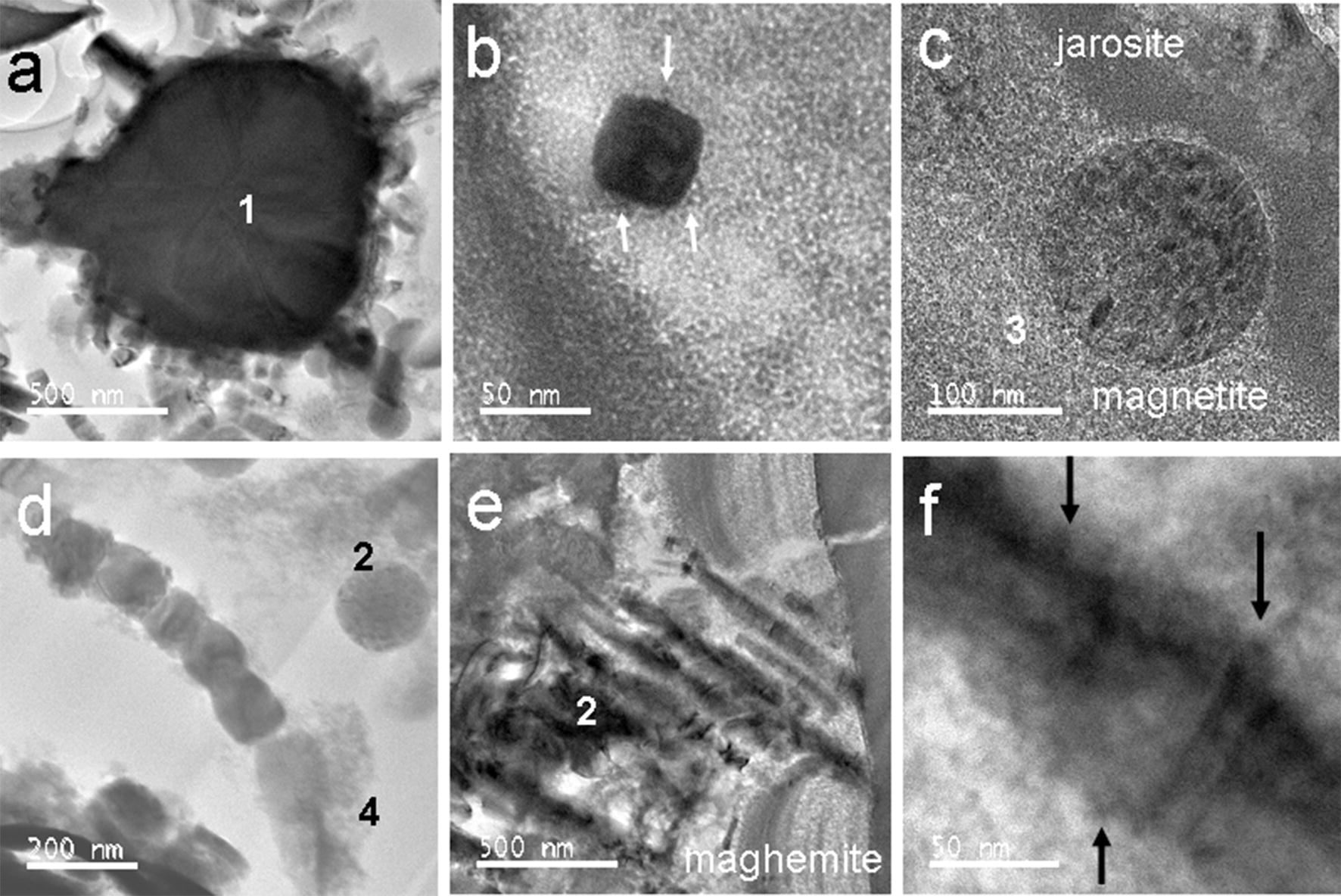
TEM images of features composed of Ni-bearing spinel-group minerals: **a**, **b** magnetite cubes; **c** spherical magnetite NP on jarosite surface; **d** row of magnetosomes; **e** parallel-growth of maghemite needles; **f** surface features on an individual maghemite needle; the presence of attached nanoparticles on the surfaces of the magnetite cube and maghemite needle are indicated with arrows in **b** and **f**; minerals are labelled with the same numbers as in Fig. 5

Mineral surface coatings are common in the upper surface layers of contaminated soils in the Sudbury area [32]. Nickel-bearing spinels such as trevorite (NiFe_2_O_4_), magnetite and maghemite (max. 1 wt% Ni) occur in different shapes and of different origins in a mineral surface coating of a Fe-rich pyroxene (Fig. 5a–f) [32, 33]. Trevorite forms a linear array of nano-domains at the interface between two zones of the mineral surface coating (Fig. 5d–f) and magnetite occurs as cubes (Fig. 6a, b), spheres (Fig. 6c) and biotic-derived lines of cubes (i.e. magnetosomes; Fig. 6d). The former magnetite crystals can be chemically altered to other Fe-(hydr)oxides such as needles of goethite (Fig. 6a) and occur either in close proximity (Fig. 5b) or are in direct contact with the surface of jarosite, KFe(SO_4_)_2_(OH)_6_ (Fig. 6c). Maghemite (identified based on morphology and supercell reflections) occurs in parallel grown needles which have been partially transformed into hematite (Fig. 6e). These mineral assemblages of nano-size crystals of Fe-(hydr)oxides (magnetite, trevorite, maghemite and goethite) and Fe-sulfates (jarosite) occur in amorphous to nano-crystalline matrices composed of silica (around jarosite) or bernalite, Fe(OH)_3_ (around the larger magnetite and maghemite crystals) [32, 33].

Crystal shapes, textures and locations of the nano-size crystals indicate that the observed spinels (magnetite, trevorite and maghemite) form during low-T abiotic and biotic dissolution of jarosite under microaerophilic conditions [32, 33]. During the latter dissolution processes, released Fe is either directly sequestered into magnetosomes (Fig. 6d) and spherical nanoparticles (Fig. 6c) or taken up by the nano-crystalline Fe-rich matrix. High resolution TEM images indicate that small nano-size particles (~ 5 nm) are attached to the outer surface of magnetite cubes (Fig. 6b) and maghemite needles (Fig. 6f). The latter feature suggests that some of the spinel nano-size crystals form at low T through crystallization by particle attachment [26] in accord with the observations on the growth of magnetite crystals through agglomeration of Fe-(hydr)oxide nanoparticles [13].

The chemical distribution map for Ni (Fig. 5c) depicts that the element is enriched in magnetite, trevorite and maghemite relative to jarosite and the Fe-rich matrix, indicating the preferential incorporation of the Ni into the former minerals [32, 33].

#### The occurrence of Zn-bearing spinels in mineralized organic matter from Trail, British Columbia, Canada

**Fig. 7:**
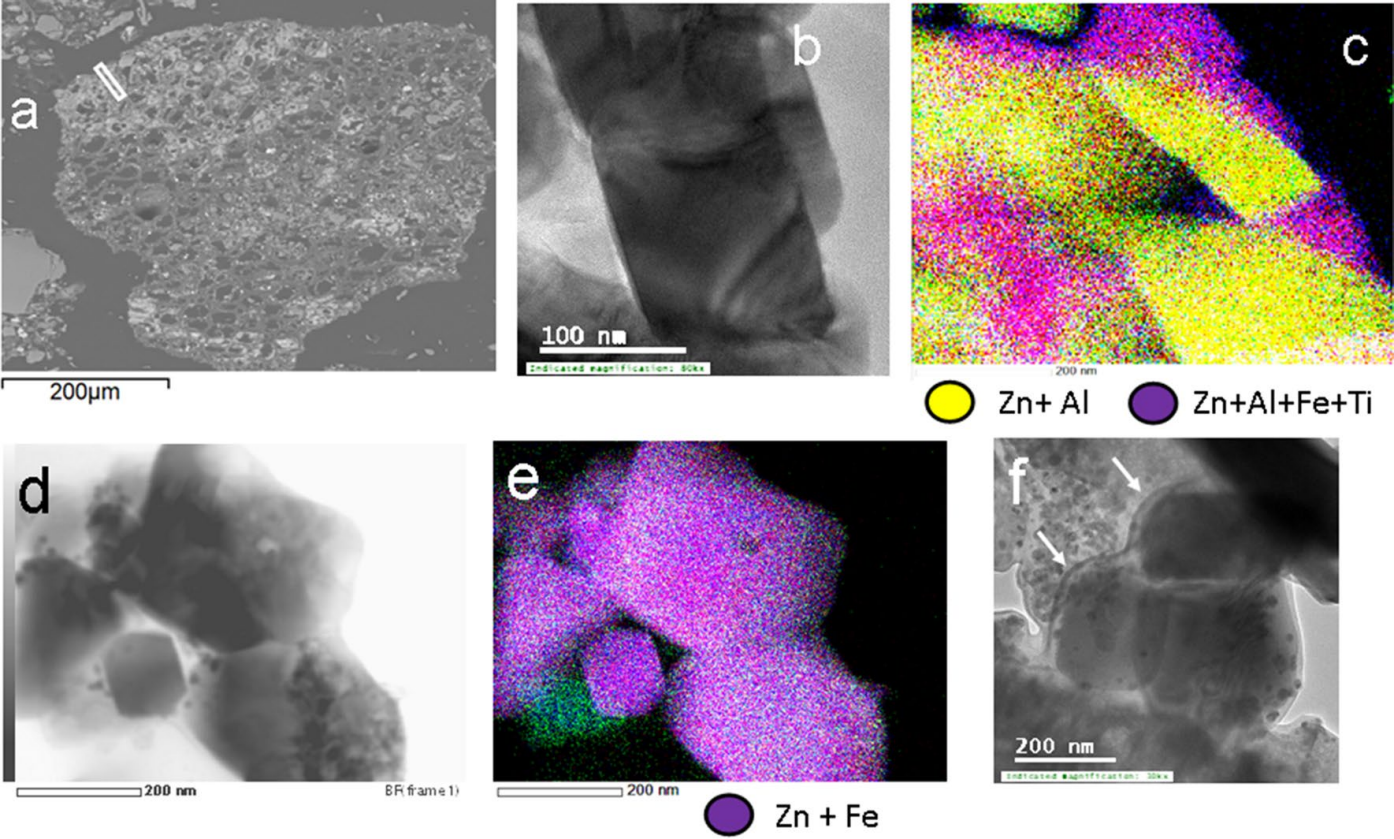
**a** Mineralized organic matter with characteristic tubular texture; area selected for FIB extraction is indicated with a white rectangular; **b**, **e** TEM and EDS-STEM chemical distribution maps of gahnite (**b**, **c**) and franklinite (**d**, **e**) crystals in the former lumina of the mineralized organic matter; **f** crystals of franklinite crystals grown parallel to lineations of organic material (indicated with arrows); colours for the elements in the chemical distribution maps are labelled accordingly

In the acidic contaminated surface layers of soils in Trail, British Columbia [36], the Zn-bearing spinels gahnite (ZnAl_2_O_4_) and franklinite (ZnFe_2_O_4_) occur in the interior of mineralized organic matter in close association with Pb–Fe-phosphates of the alunite group and anglesite [35] (Fig. 7). Gahnite occurs as euhedral blocky crystals (Fig. 7b) which have been partially altered by a Zn–Al–Fe-Ti rich hydroxide phase (Fig. 7c). Franklinite crystals can occur either as idiomorphic octahedra (Fig. 7d, e), cuboctahedra or cubes (Fig. 7f). The latter idiomorphic crystals are often aligned parallel to mineralized lineations of organic material (labelled with arrows in Fig. 7f). Similar to the occurrence of spinel-group minerals in the dendrites of the Pb-bearing silica glass and in the mineral surface coatings of the Sudbury area, euhedral crystals and growth features of the Zn-bearing spinel group minerals point towards their formation in the mineralized organic matter under ambient Earth surface conditions [35]. This conclusion is in accordance with previous observations from the low-T formation of gahnite in floodplain sediments (mine tailings) from the Clark Fork River Superfund Site in Montana, USA [37].

#### The occurrence of Zn-Sb-bearing spinels in mineral surface coatings from Trail, British Columbia, Canada

**Fig. 8:**
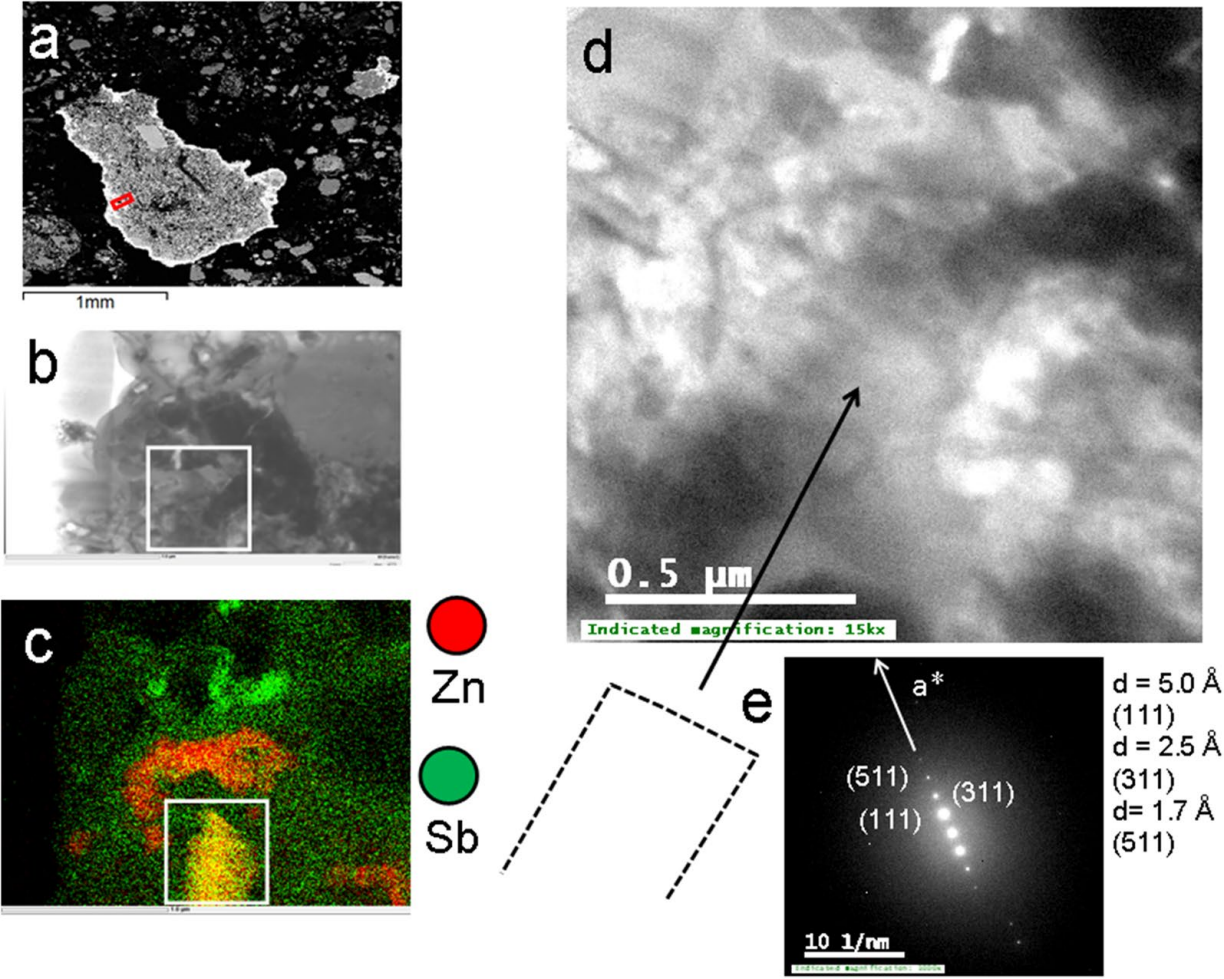
**a** Selected mineral surface coatings on an agglomerate of mainly Ca–Mg rich amphibole grains, the area selected for FIB extraction is indicated with a red rectangular; **b**, **c** TEM and EDS-STEM images of an area containing a Zn-Fe-Sb spinel; **d** TEM image of an elongated prismatic Zn-Fe-Sb spinel crystal; the outline of the crystal is depicted below the image; **e** selected area diffraction pattern indicating well define diffractions spots along the reciprocal a* axis; colours of the elements in the chemical distribution map and d-spacings and (hkl) indices are labelled accordingly in **c** and **e**

A spinel-type phase with a Zn:Fe:Sb ratio of 7:4:2 occurs within a mineral surface coatings in the upper surface layers of the acidic soils in Trail, British Columbia (Fig. 8a) [35]. The spinel crystal is embedded in an amorphous silica matrix (Fig. 8b, c) as a relatively large elongated prism (~ 0.5 µm along the length axis, Fig. 8d, e). Zinc-antimony spinels such as Zn_2.33_Sb_0.67_O_4_ (cubic) and Zn_7_Sb_2_O_12_ (orthorhombic) present a group of spinel phases in which Sb occurs in its pentavalent state and which can be synthesized in high-T sintering processes above T = 900 °C [38]. The euhedral shape of the observed crystal in the mineral surface coatings indicates however that Zn–Sb spinels can also form under ambient Earth surface conditions.

## Conclusions and implications

Nano-size crystals of spinel group minerals are previously unknown features in low-T alteration products such as dendritic growth features, mineral surface coatings and mineralized organic matter. These euhedral crystals and absence of any cooling or alteration features indicate the formation of these minerals under ambient Earth surface conditions rather than their formation under high T processes and their subsequent reworking through fluvial or Aeolian processes. All the observed nano-size crystals of spinel group minerals have been identified in confined pore spaces which suggest that their euhedral formation may be promoted by limitations in the diffusive transport of solutes or solvents to the respective pore space [39].

Charge-balance mechanisms and the occurrence of two distinct cation coordination sites allows the spinel structure to sequester the major contaminants Ni^2+^, Zn^2+^ and Sb^5+^ in the surficial soils of the Timmins, Sudbury, and Trail areas. The formation of contaminant-bearing nano-size crystals of spinels rather than more soluble hydroxide and oxy-salt minerals under ambient Earth surface conditions indicate that contaminants compatible with the spinel-structure type can be sequestered through geochemical soils processes in these low-soluble minerals within surficial soils. The weathering resistance of the spinel group minerals formed in low-T environments may subsequently allow the transport of the sequestered contaminants over extended distances by alluvial or Aeolian processes. However, as far as we know and to quantify this further, the dissolution rates of nano-spinels (vs. bulk spinels) need to be measured. It is important to do so, as it is possible, but not certain, that dissolution rates will increase dramatically in the nanoscale size range [40].

## Additional file

**Additional file 1.** Additional optical microscope-, SEM- and TEM images, chemical analyses, selected area electron diffraction pattern with d-spacings and FFT analyses of lattice fringes with d-spacings.

## Abbreviations

T: temperature
SEM: scanning electron microscope
TEM: transmission electron microscope
EDS: energy dispersive spectroscopy
NPs: nanoparticles
PM: particulate matter
FIB: focused ion beam
STEM: scanning transmission electron microscopy
BF: bright field
SAED: selected area electron diffraction
FFT: fast Fourier transformation
CPA: crystallization through particle attachment
