# Supplementary material for: The formation of spinel-group minerals in contaminated soils: the sequestration of metal(loid)s by unexpected incidental nanoparticles
Source: Geochem Trans. 2019 Mar 13;20:1. doi: 10.1186/s12932-019-0061-3 (PMC6743039; doi:10.1186/s12932-019-0061-3)
Supplement: Supplementary file 1 — Additional file 1. Additional optical microscope-, SEM- and TEM images, chemical analyses, selected area electron diffraction pattern with d-spacings and FFT analyses of lattice fringes with d-spacings. [file 12932_2019_61_MOESM1_ESM.pdf]

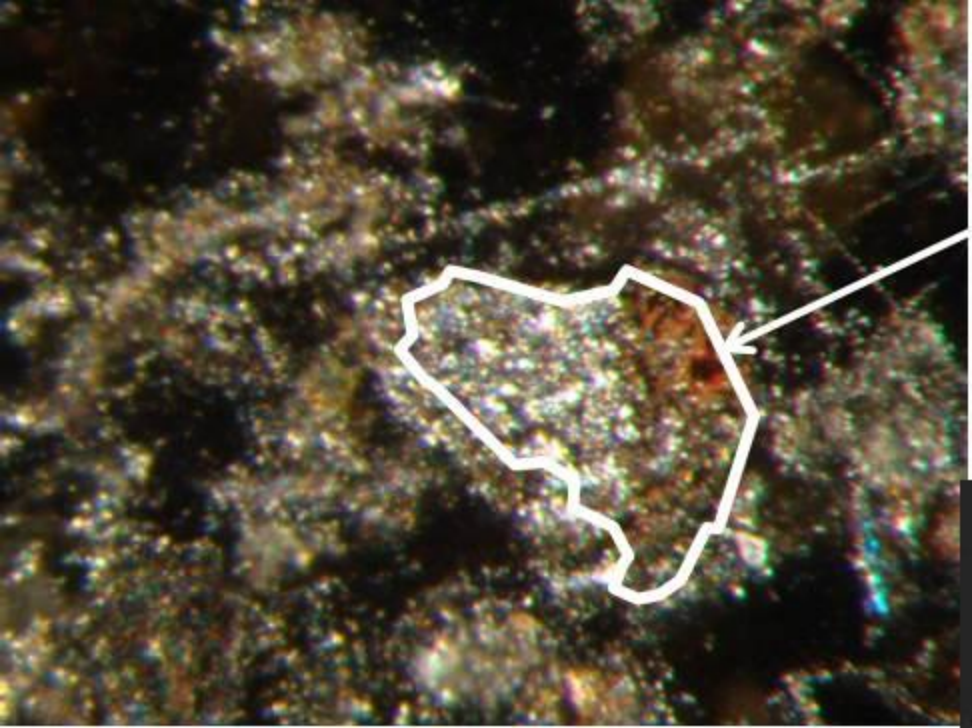

Red alteration rim

S1
Pb-silica glass grain

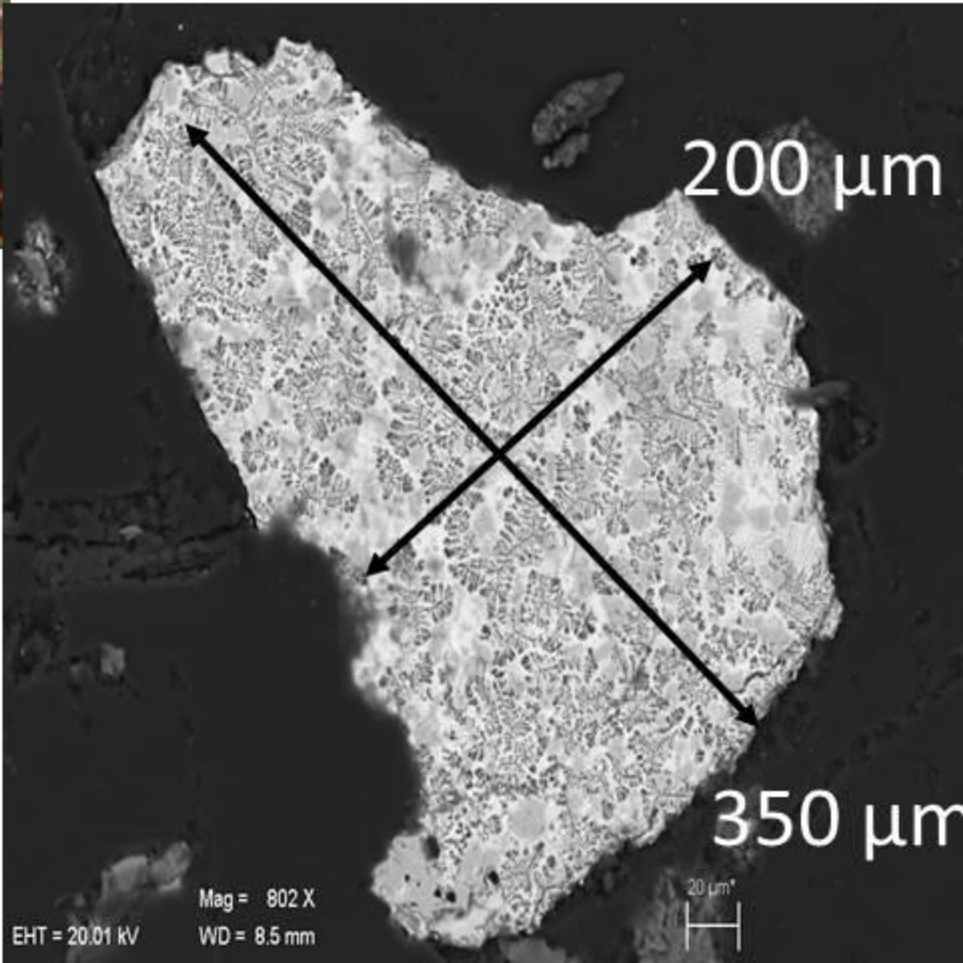

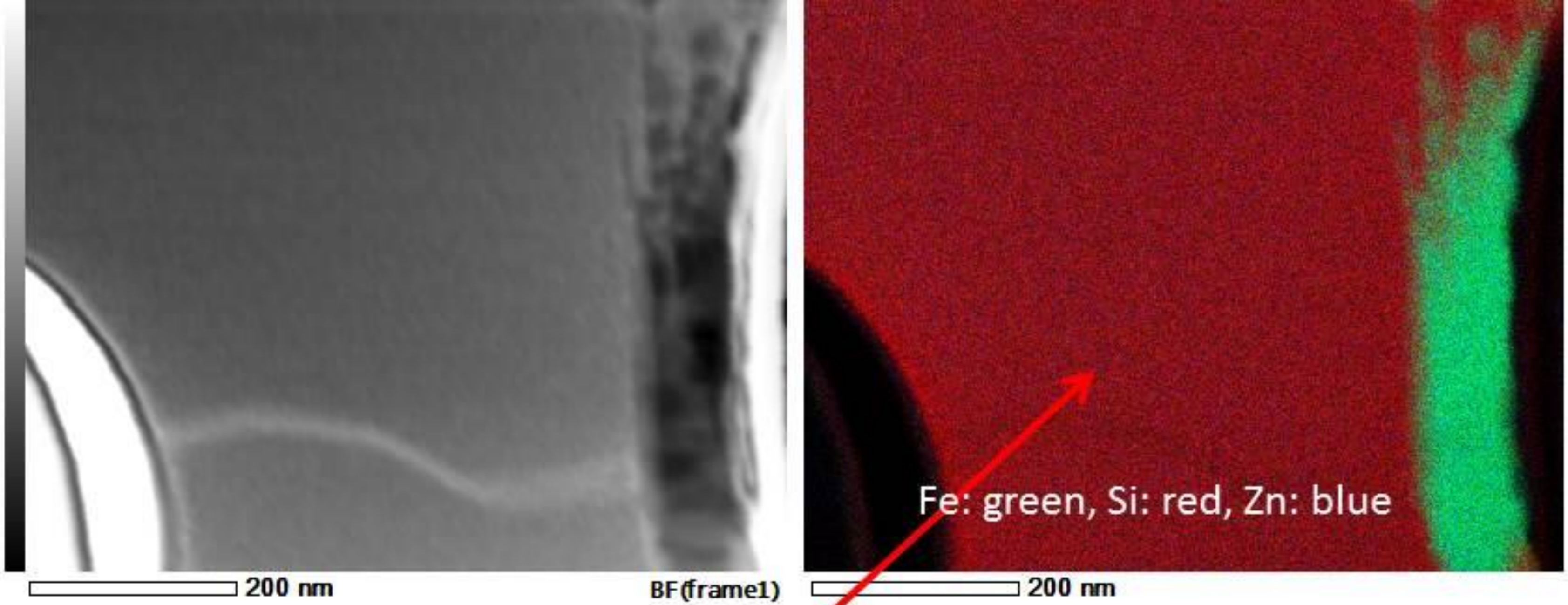

Element	(keV)	Counts	Mass%	Sigma	Atom%
Mg K	1.253	4339.61	0.95	0.03	2.62
Al K	1.486	6341.05	0.71	0.02	1.77
Si K	1.739	113780	26.18	0.12	62.25
K K	3.312	1536.60	0.40	0.02	0.68
Ca K	3.690	5477.23	1.51	0.03	2.52
Fe K	6.398	15216	5.55	0.06	6.64
Cu K	8.040	6299.20	1.58	0.03	1.66
Zn K	8.630	5450.40	2.17	0.04	2.22
Pb M	2.342	57018	60.93	0.37	19.64
Total			100.00		100.00

S2 chemical analysis of unaltered glass matrix

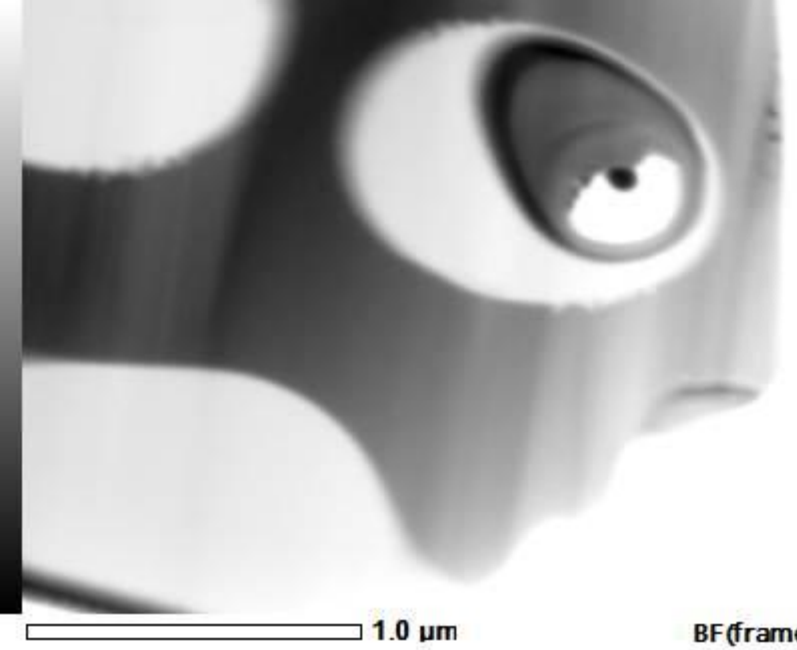

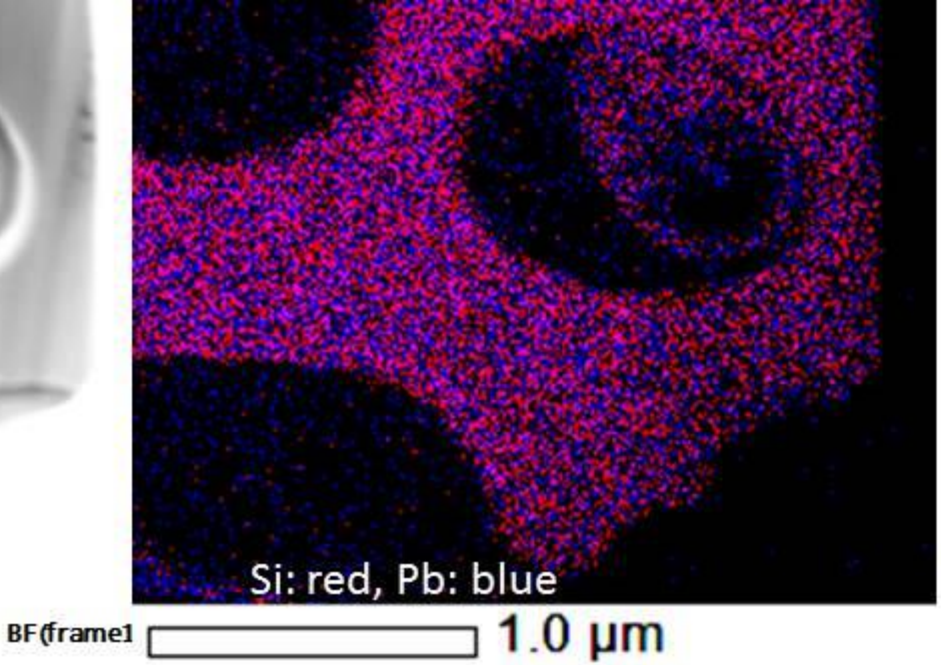

Element		(keV)	Counts	Mass%	Sigma	Atom%
Mg K	!	1.253	2939.39	1.10	0.04	3.14
Si K		1.739	59845	23.40	0.15	57.94
Al K	!	1.486	3310.51	0.63	0.02	1.63
K K	!	3.312	588.60	0.26	0.02	0.46
Ca K	!	3.690	3534.04	1.66	0.04	2.88
Fe K		6.398	11702	7.25	0.09	9.03
Cu K	!	8.040	2891.58	1.23	0.04	1.35
Zn K	!	8.630	3894.61	2.64	0.06	2.81
Pb M		2.342	34042	61.82	0.49	20.75
Total				100.00		100.00

S3 chemical analysis of unaltered glass matrix

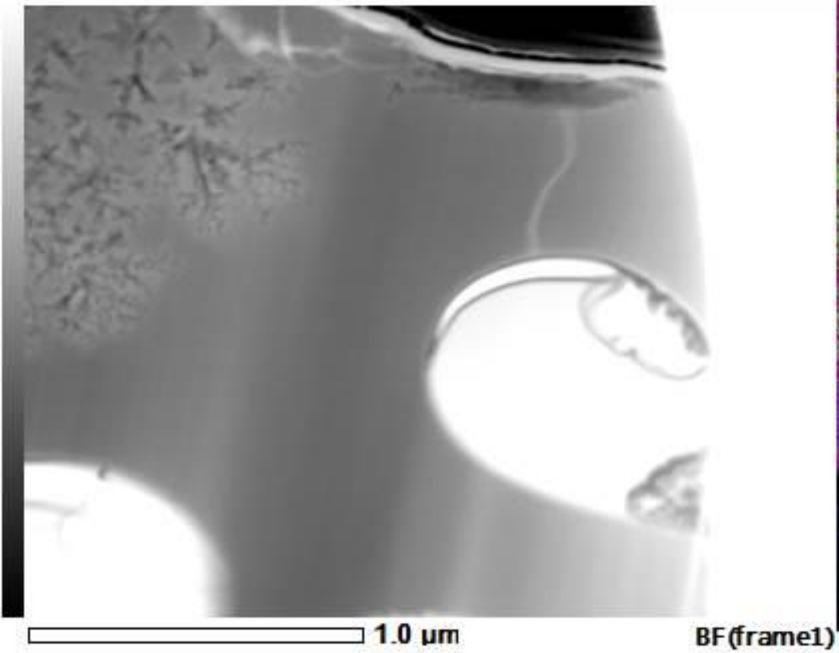

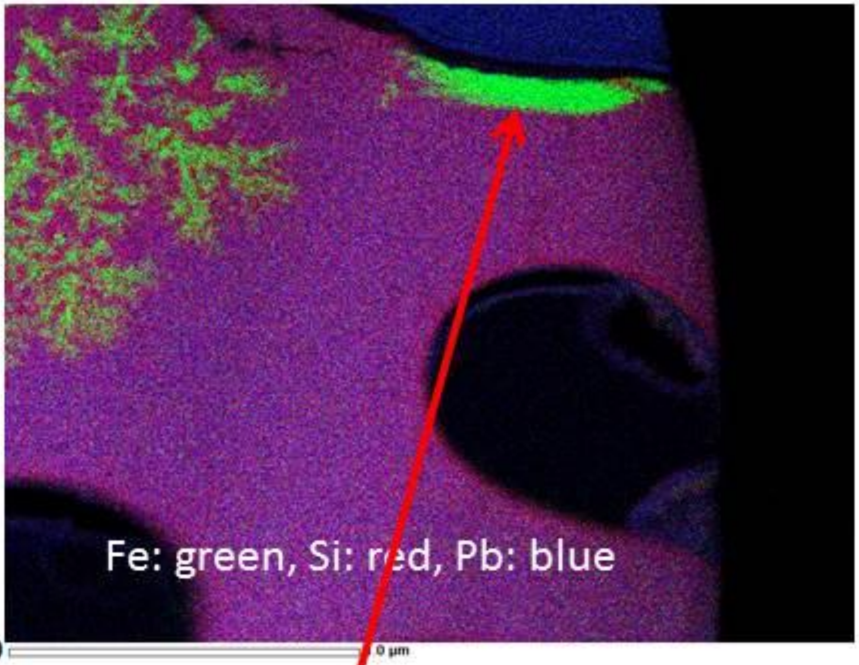

Element	(keV)	Counts	Mass%	Sigma	Atom%
Al K	1.486	775.59	0.30	0.02	0.67
Si K	1.739	3986.27	1.51	0.03	3.19
Fe K	6.398	121226	72.57	0.10	77.22
Cu K	8.040	2544.04	1.94	0.03	1.82
Zn K	8.630	19929	16.60	0.08	15.08
Pb M	2.342	4035.85	7.08	0.22	2.03
Total			100.00		100.00

S4 chemical analysis of remaining of large Zn-rich magnetite crystal

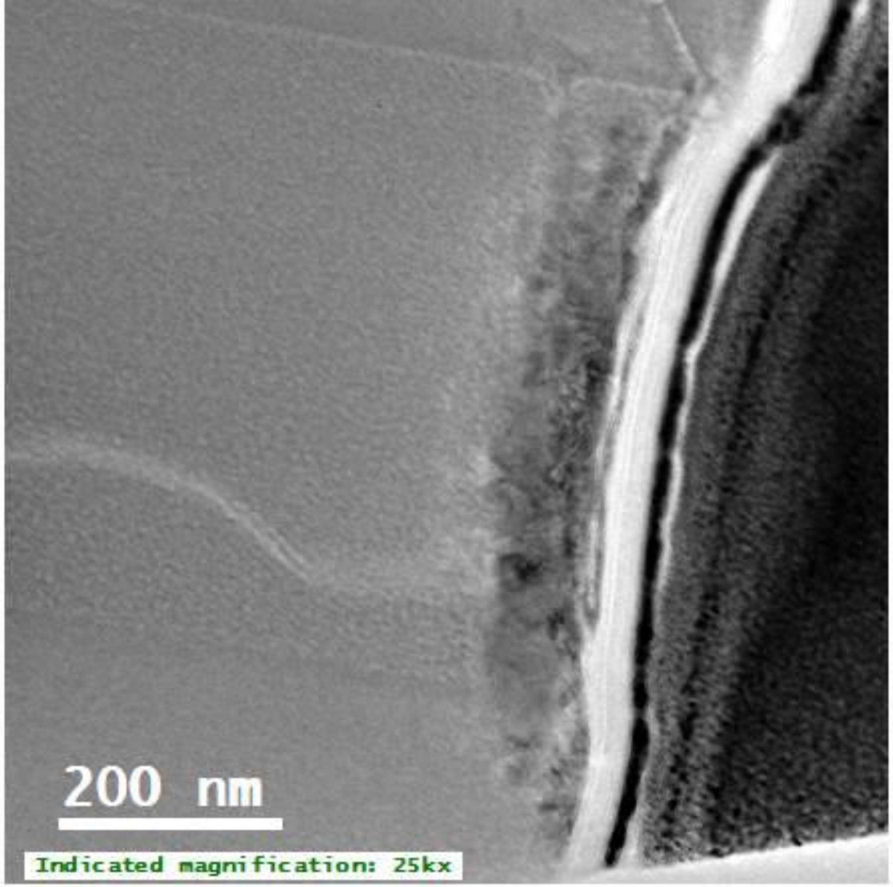

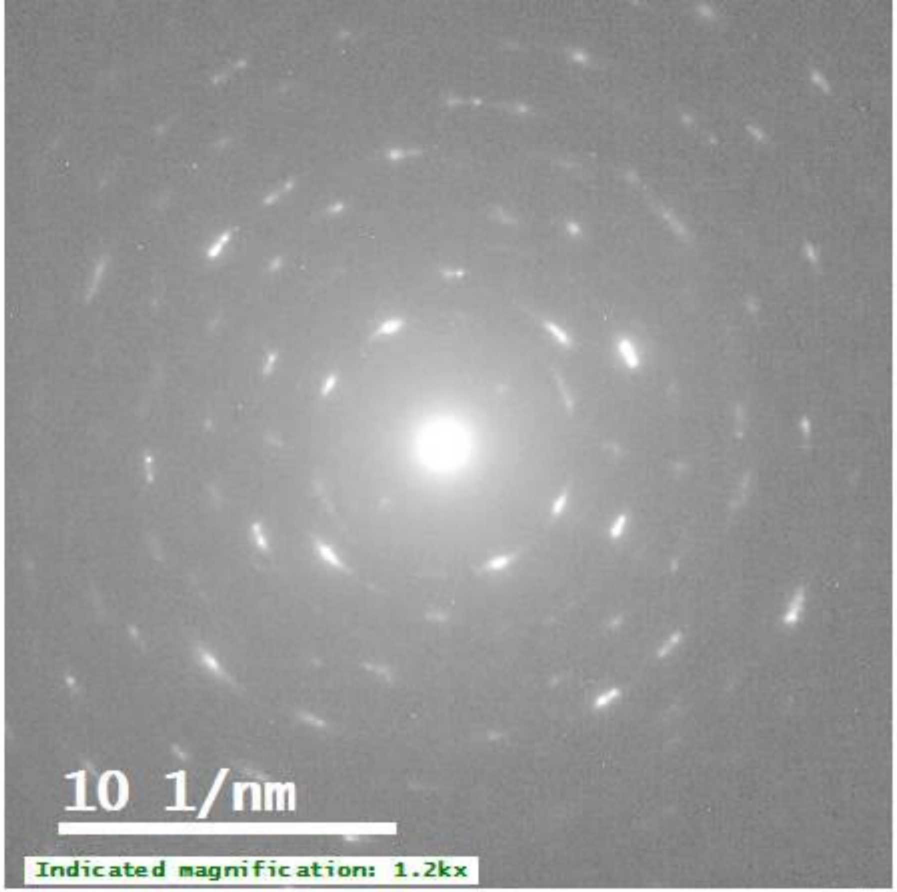

S5 Selected Area Electron Diffraction
of the remaining of a micrometer-size
Zn-rich magnetite crystal $(Zn_{0.5}Fe^{2+}_{0.5})Fe^{3+}_2O_4$
Magnetite: 2.55 (311), 2.10 (400)
Traces of goethite: d = 4.1 (101), 1.91 (410)

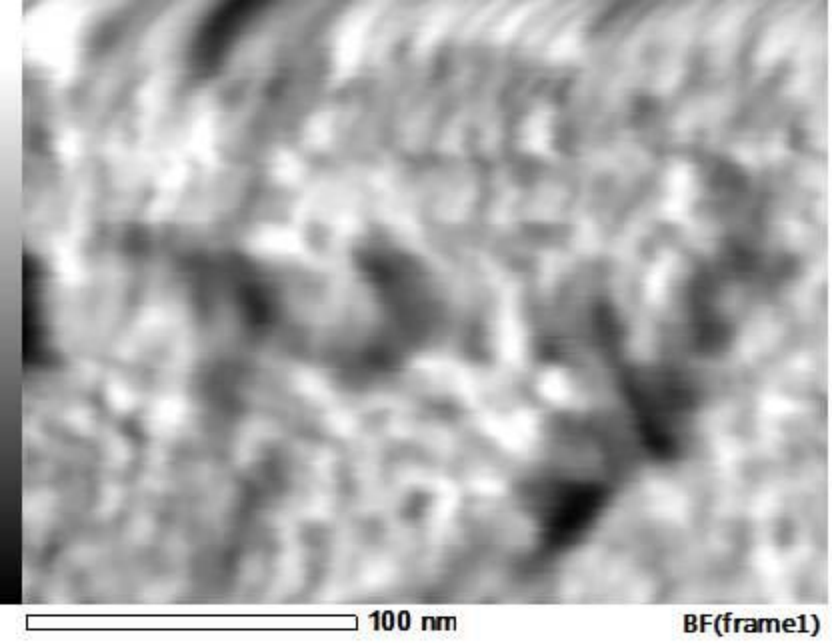

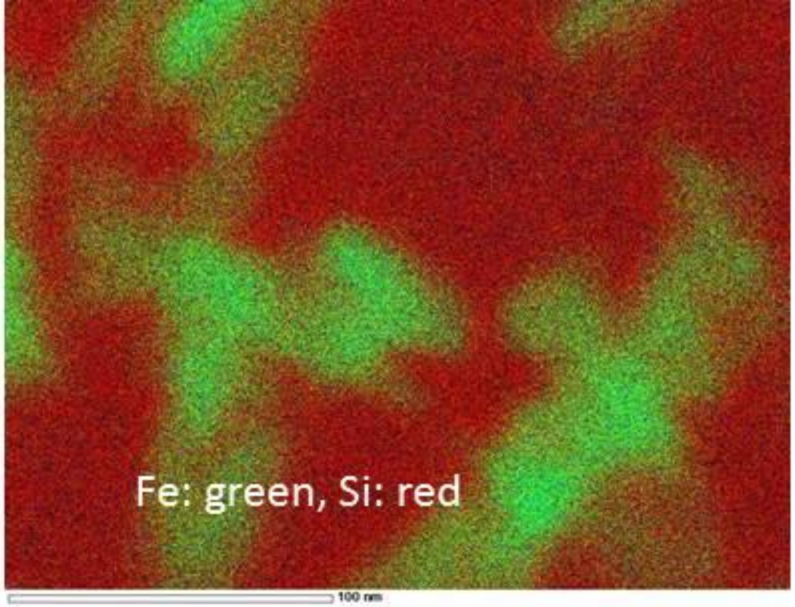

Element	(keV)	Counts	Mass%	Sigma	Atom%
Al K	1.486	1203.50	0.89	0.05	1.80
Si K	1.739	26436	18.82	0.18	36.56
Ca K	3.690	681.10	0.56	0.04	0.77
Fe K	6.398	40534	45.74	0.31	44.69
Cu K	8.040	1146.21	1.65	0.08	1.42
Zn K	8.630	6972.49	10.95	0.19	9.13
Pb M	2.342	6465.94	21.38	0.46	5.63
Total			100.00		100.00

S6 chemical analysis of Green areas with contributions of red area

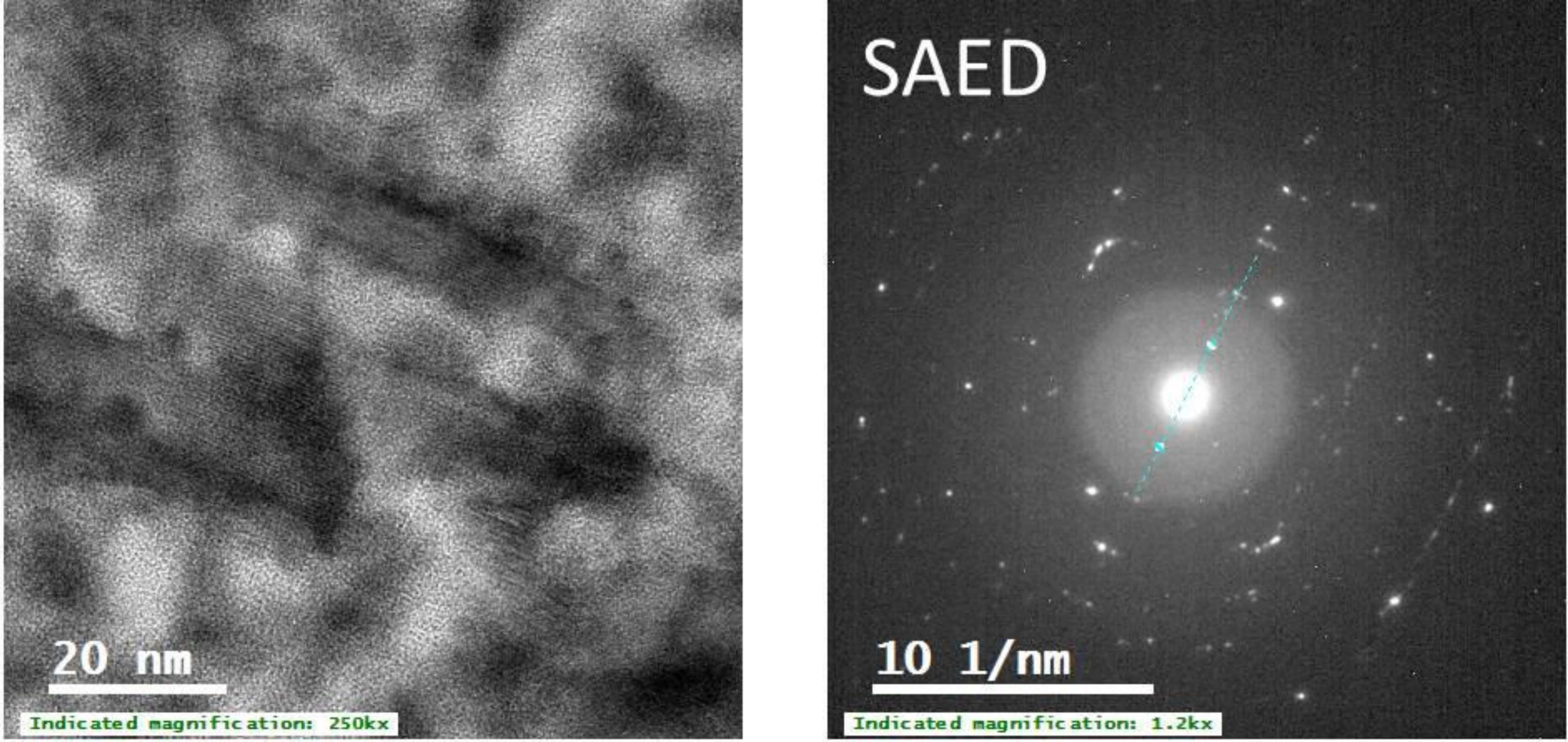

S7 Selected Area Electron Diffraction of Zn-rich magnetite

D=4.87 (111), D=2.50 (311), D=2.09 Å (400)

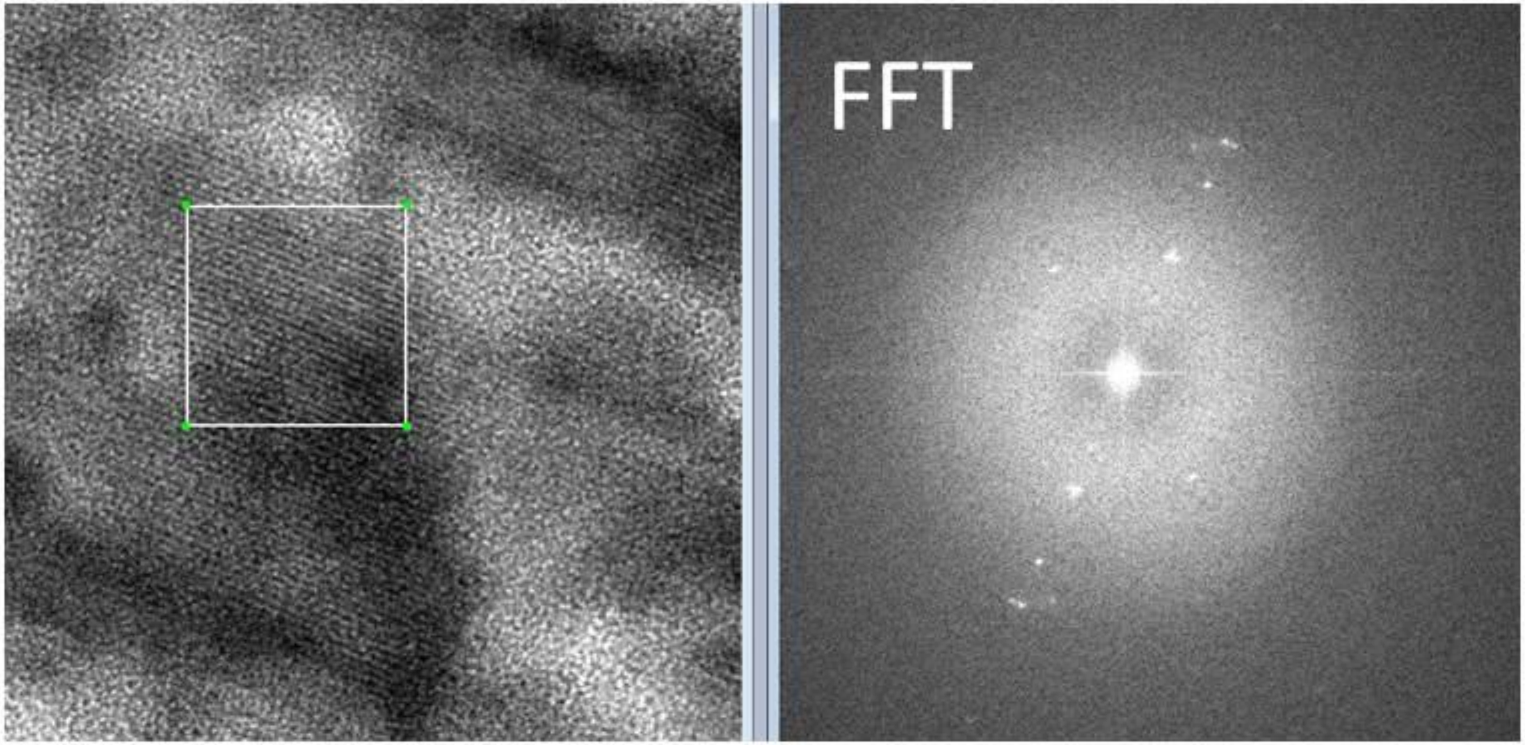

S8

FFT of Zn-rich spinel

D=4.8 (111), 2.9 (220), 2.5 (311) Å

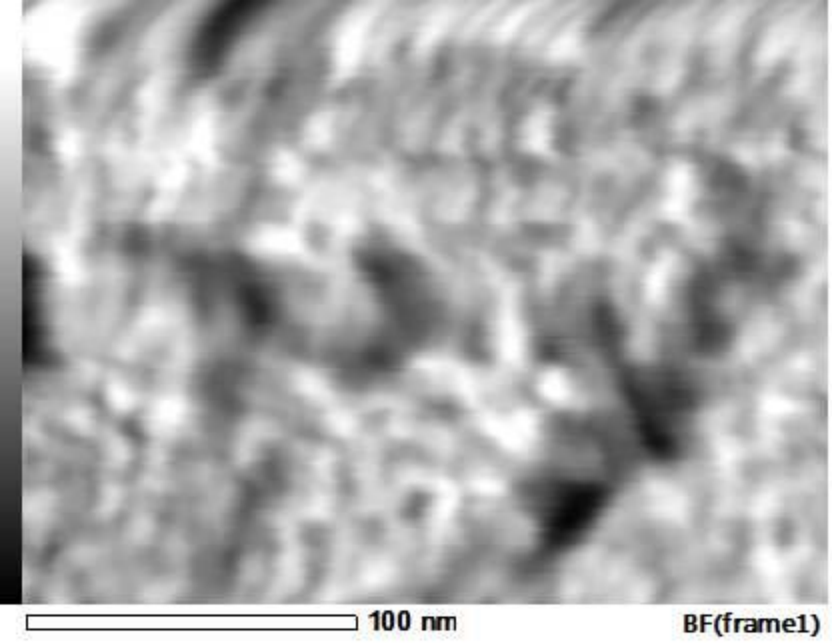

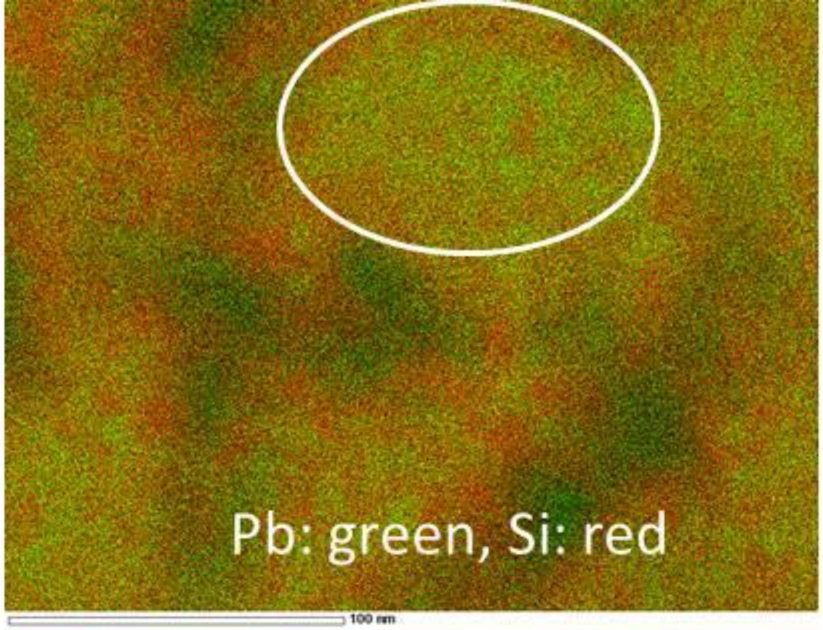

Element	(keV)	Counts	Mass%	Sigma	Atom%
Al K	1.486	1643.48	0.90	0.04	2.14
Si K	1.739	26653	29.84	0.29	68.19
Ca K	3.690	1142.43	1.54	0.07	2.46
Fe K	6.398	2641.65	4.69	0.13	5.39
Cu K	8.040	1105.42	1.35	0.06	1.36
Zn K	8.630	1034.81	2.01	0.10	1.97
Pb M	2.342	11474	59.67	0.83	18.48
Total			100.00		100.00

S9 chemical analysis of Minium Pb3O4 nanoparticles (encircled)

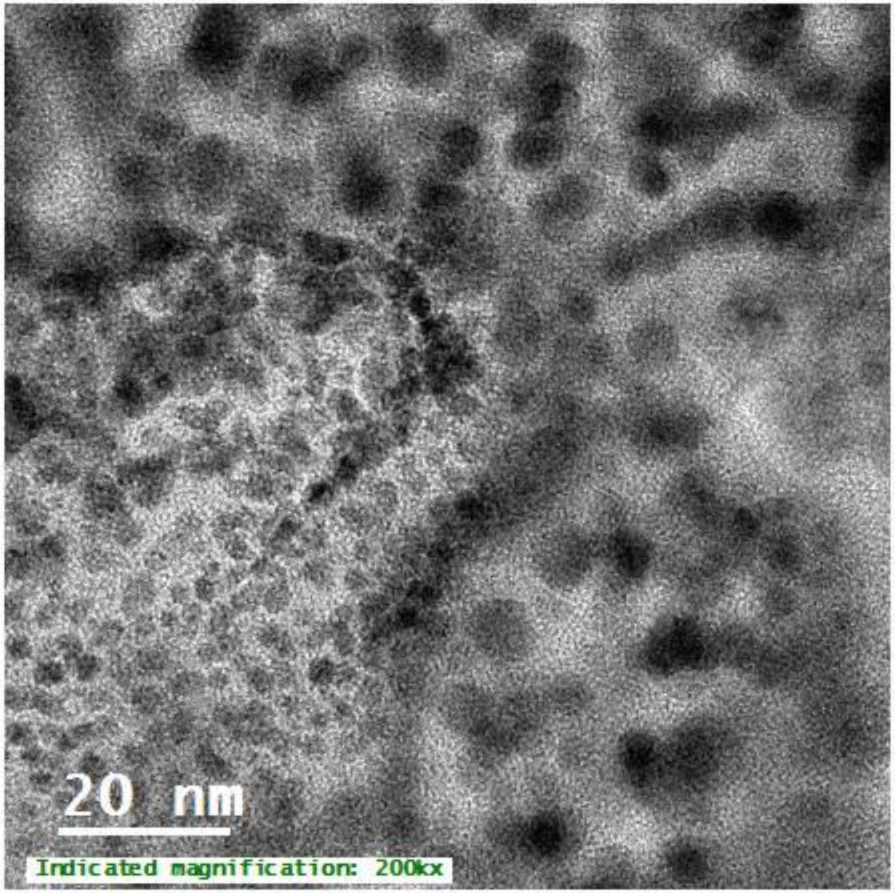

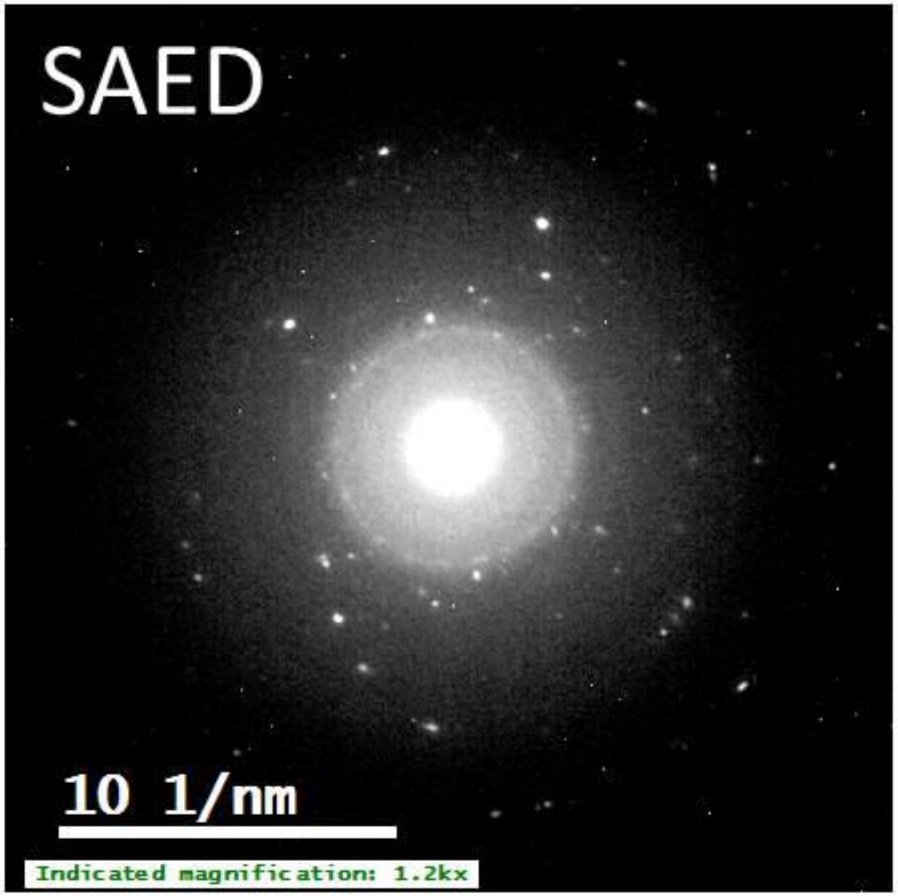

S10

Diffuse rings or spots: minium, d = 4.0 (210), 2.82 (310), 2.25 (222)

Sharp spots: Zn-rich magnetite, d= 2.55 (311), 2.10 (400)

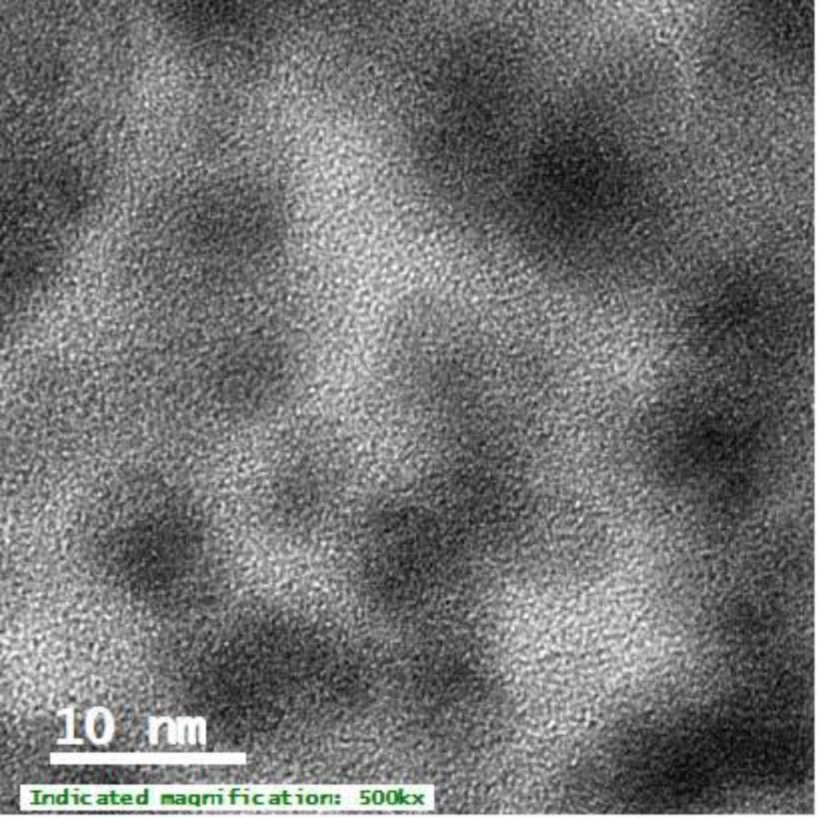

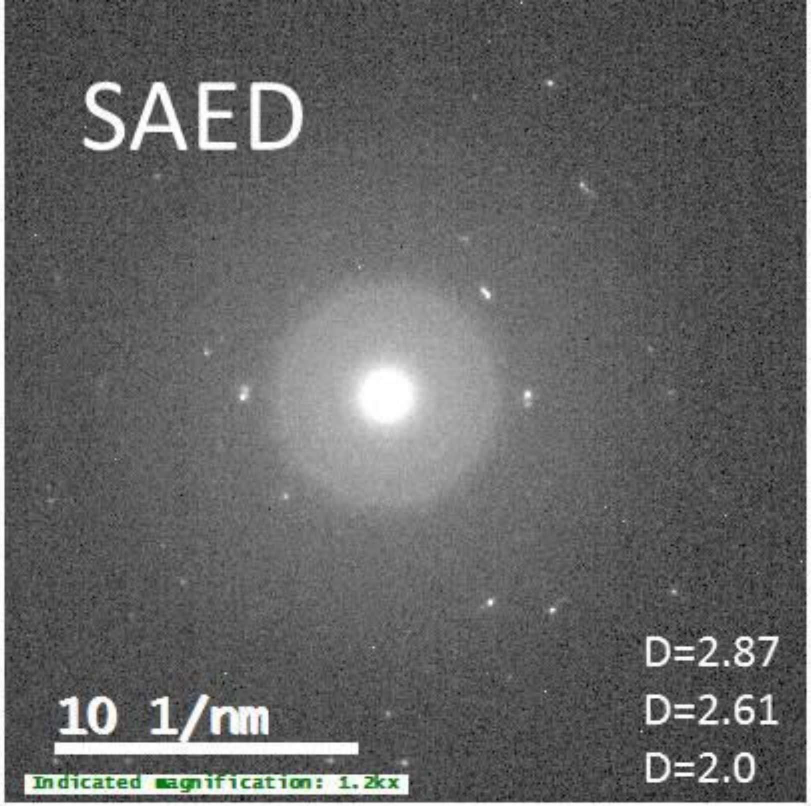

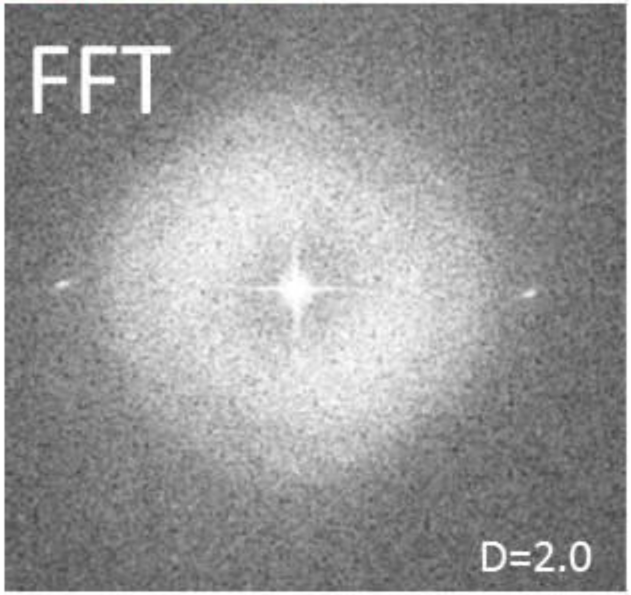

S11
Identification of minium nanoparticles

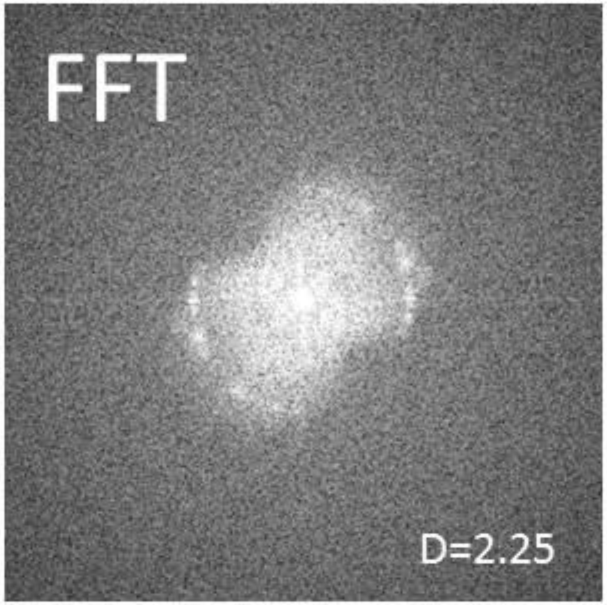

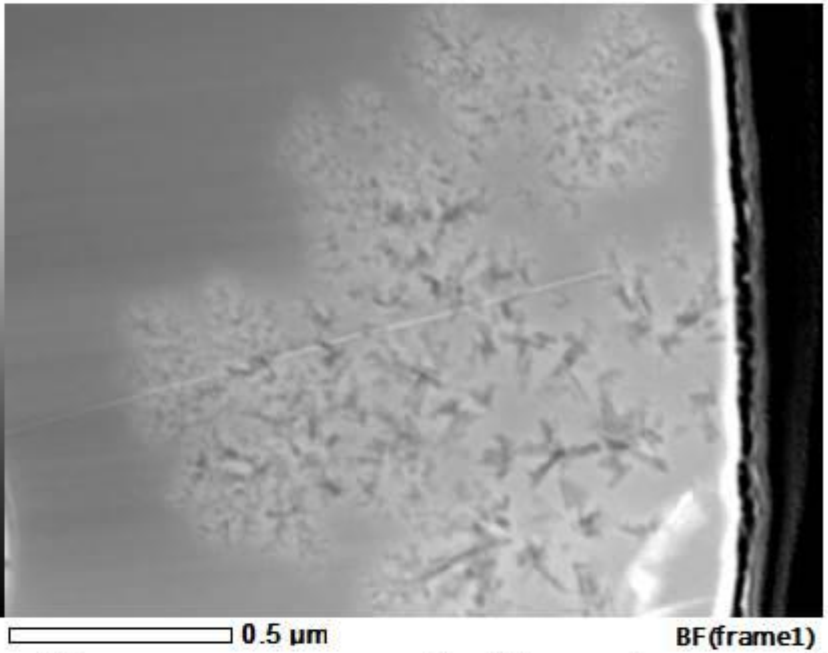

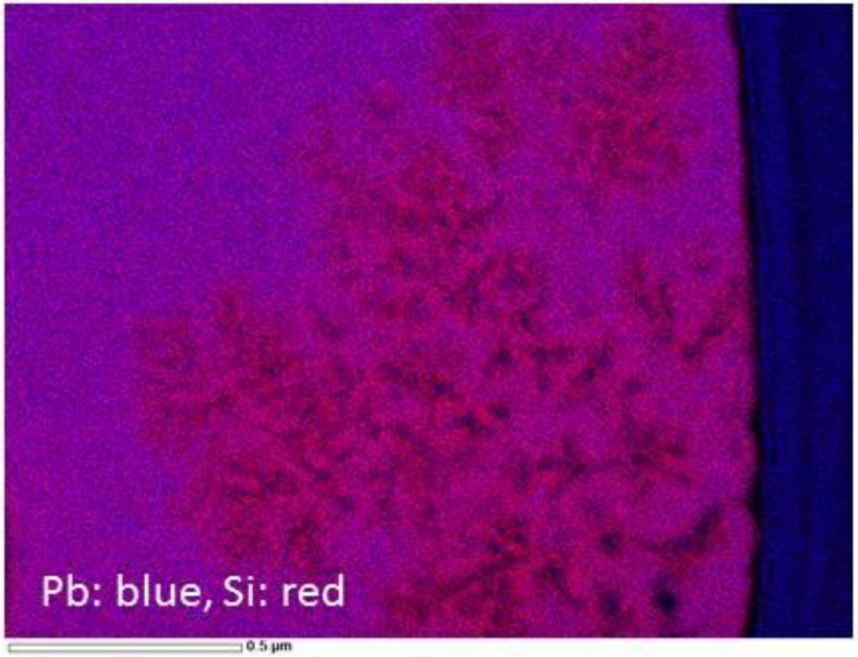

Element	(keV)	Counts	Mass%	Sigma	Atom%
Mg K	1.253	34.01	0.47	0.15	0.98
Al K	1.486	128.61	0.91	0.11	1.71
Si K	1.739	2729.36	39.61	0.71	71.36
Ca K	3.690	36.44	0.63	0.22	0.80
Fe K	6.398	578.65	13.31	0.41	12.06
Cu K	8.040	44.06	0.70	0.27	0.56
Zn K	8.630	127.35	3.20	0.41	2.48
Pb M	2.342	610.76	41.16	1.93	10.05
Total			100.00		100.00

S12 Chemical composition of Si-rich areas (pink) between dendrites (black) and unalered glass (violet)
